# Visualization of myelin‐forming oligodendrocytes in the adult mouse brain

**DOI:** 10.1111/jnc.16218

**Published:** 2024-09-04

**Authors:** Kiichi Yokoyama, Yuichi Hiraoka, Yoshifumi Abe, Kenji F. Tanaka

**Affiliations:** ^1^ Division of Brain Sciences Institute for Advanced Medical Research, Keio University School of Medicine Tokyo Japan; ^2^ Laboratory of Molecular Neuroscience Medical Research Institute, Tokyo Medical and Dental University Tokyo Japan; ^3^ Laboratory of Genome Editing for Biomedical Research Medical Research Institute, Tokyo Medical and Dental University Tokyo Japan

**Keywords:** differentiation, long noncoding RNA, newly formed oligodendrocyte, oligodendrocyte, oligodendrogenesis, premyelinating oligodendrocyte

## Abstract

Oligodendrocyte (OL) differentiation from oligodendrocyte precursor cells (OPCs) is considered to result in two populations: premyelinating and myelinating OLs. Recent single‐cell RNA sequence data subdivided these populations into newly formed (NFOLs), myelin‐forming (MFOLs), and mature (MOLs) oligodendrocytes. However, which newly proposed population corresponds to premyelinating or myelinating OLs is unknown. We focused on the NFOL‐specific long non‐coding oligodendrocyte 1 gene (*LncOL1*) and sought to label NFOLs under the control of the *LncOL1* promoter using a tetracycline‐controllable gene induction system. We demonstrated that *LncOL1* was expressed by premyelinating OLs and that the MFOL‐specific gene, *Ctps*, was not, indicating that NFOLs correspond to premyelinating OLs and that MFOLs and MOLs correspond to myelinating OLs. We then generated a *LncOL1*‐tTA mouse in which a tetracycline transactivator (tTA) cassette was inserted downstream from the *LncOL1* transcription initiation site. By crossing the *LncOL1*‐tTA mice with tetO reporter mice, we generated *LncOL1*‐tTA::tetO‐yellow fluorescent protein (YFP) double‐transgenic (*LncOL1*‐YFP) mice. Although *LncOL1* is non‐coding, YFP was detected in *LncOL1*‐YFP mice, indicating successful tTA translation. Unexpectedly, we found that the morphology of *LncOL1*‐tTA‐driven YFP^+^ cells was distinct from that of *LncOL1* ^+^ premyelinating OLs and that the labeled cells instead appeared as myelinating OLs. We demonstrated from their RNA expression that YFP‐labeled OLs were MFOLs, but not MOLs. Using the unique property of delayed YFP induction, we sought to determine whether MFOLs are constantly supplied from OPCs and differentiate into MOLs, or whether MFOLs pause their differentiation and sustain this stage in the adult brain. To achieve this objective, we irradiated adult *LncOL1*‐YFP brains with X‐rays to deplete dividing OPCs and their progeny. The irradiation extinguished YFP‐labeled OLs, indicating that adult OPCs differentiated into MOLs during a single period. We established a new transgenic mouse line that genetically labels MFOLs, providing a reliable tool for investigating the dynamics of adult oligodendrogenesis.

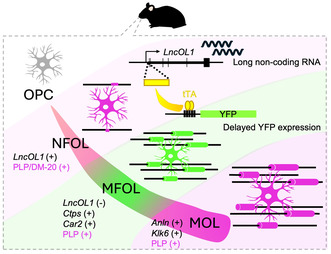

## Graphical abstract

Single‐cell RNA sequence data subdivided oligodendrocyte differentiation steps into newly formed oligodendrocytes (NFOLs), myelin‐forming oligodendrocytes (MFOLs), and mature oligodendrocytes (MOLs). We focused on NFOL‐specific RNA, long non‐coding oligodendrocyte 1 (*LncOL1*) and generated an *LncOL1*‐tTA mouse line. By crossing the line with the tetO reporter line, we obtained the *LncOL1*‐tTA::tetO‐YFP double transgenic mice. We unexpectedly found that the *LncOL1*‐tTA‐driving YFP^+^ cells were depicted as myelinating OLs. We further demonstrated that YFP‐labeled OLs were MFOLs and that NFOLs and MFOLs were consecutive and transient population in the adult brain.

**Abbreviations**
*Anln*
: anillin
BAC: bacterial artificial chromosome
BCIP: 5‐bromo‐4‐chloro‐3‐indolyl phosphate
*Car2*
: carbonic anhydrase 2
CRISPR/Cas‐9: clustered regularly interspaced short palindromic repeats/CRISPR associated proteins 9
*Ctps*
: cytidine 5′‐triphosphate synthetase
DAPI: 4,6‐diamidino‐2‐phenylindole
DIG: digoxigenin
DNA: deoxyribonucleic acid
FISH: fluorescence in situ hybridization
GFP: green fluorescent protein
ISH: *in situ* hybridization
*Klk6*
: kallikrein 6
*LncOL1*
: long non‐coding oligodendrocyte 1
*MALAT*
: metastasis‐associated lung adenocarcinoma transcript 1
MFOL: myelin‐forming oligodendrocyte
MOL: mature oligodendrocyte
*mtTA*
: mammalianized tTA
NBT: nitro blue tetrazolium
NFOL: newly formed oligodendrocyte
NG2: neuron‐glial antigen 2
OL: oligodendrocyte
OPC: oligodendrocyte precursor cells
PB: phosphate buffer
PBS: phosphate‐buffered saline
PCR: polymerase chain reaction
*Pdgfra*
: platelet‐derived growth factor receptor alpha
PFA: paraformaldehyde
PLP: proteolipid protein
qRT‐PCR: quantitative reverse transcription PCR
RNA: ribonucleic acid
ROI: region of interest
RRID: Research Resource Identifier (see scicrunch.org)
SV 40: simian virus 40
tet‐system: tetracycline‐controllable gene induction system
tTA: tetracycline transactivator
YC: yellow cameleon
YFP: yellow fluorescent protein

## 1 INTRODUCTION

Recent single‐cell RNA sequencing revealed oligodendrocyte (OL) heterogeneity in terms of gene expression and categorized OL‐lineage cells into subclasses (Figure S1; Marques et al., 2016). Among them, newly formed oligodendrocytes (NFOLs) and myelin‐forming oligodendrocytes (MFOLs) presumably represent a population intermediate between OL precursor cells (OPCs) and mature OLs (MOLs). Although single‐cell RNA sequencing is a powerful tool for examining gene expression on a single‐cell basis, it does not capture the actual morphology of each cell.

The morphology of OLs is an indicator of their differentiation state. Historically, OLs have been categorized into three groups according to their unique protein expression and morphology: OPCs, premyelinating OLs, and myelinating OLs (Trapp et al., 1997). Both OPCs and premyelinating OLs radially extend ramified and elaborate processes, while myelinating OLs are rather characterized by parallel processes associated with myelinated axons (Nishiyama et al., 2002; Trapp et al., 1997). OPCs and premyelinating OLs can be distinguished by neuron‐glial antigen 2 (NG2) and proteolipid protein/DM‐20 (PLP/DM‐20), respectively (Nishiyama et al., 2002; Trapp et al., 1997). Because transcriptional and morphological classifications were independently proposed, the correspondence between these two has been poorly understood. We sought to fill this gap between the classifications by visualizing OLs in the process of differentiation with a reliable genetic tool.

The long non‐coding oligodendrocyte 1 gene (*LncOL1*) is a promising marker for NFOLs. Zhang et al. (2014) first reported that the RNA *9630013A20Rik* was highly expressed in NFOLs. Later, He et al. (2017) presented the epigenetic landscape of the OLs and named the RNA sequence “*LncOL1*.” Concurrently, single‐cell analysis of juvenile mouse brains by Marques et al. (2016) revealed that high *LncOL1* expression was confined to the NFOL population, with other OL subsets showing only lower expression. These single‐cell RNA sequencing data lack information on cellular morphology and differentiation time scales, leaving questions such as (1) whether NFOLs that are defined by gene expression correspond to premyelinating OLs defined by morphology and (2) whether, in the adult brain, NFOLs are continuously supplied from dividing OPCs and differentiating to MFOLs, or whether NFOL production and differentiation occur only in the developing brain, where their differentiation is arrested and their gene profile is maintained throughout life.

In the present study, we sought to determine whether *LncOL1* ^+^ NFOLs correspond to premyelinating OLs. We then sought to label NFOLs genetically to visualize their morphology. We selected a tetracycline‐controllable gene induction system (tet‐system) to achieve this objective. Compared with indelible labeling using a Cre‐loxP system, the tet‐system targets cells only during tTA expression and excels in temporal monitoring of gene expression, such as that by *LncOL1*.

## 2 MATERIALS AND METHODS

### 2.1 Animals

All animal procedures were conducted in accordance with the National Institutes of Health Guide for the Care and Use of Laboratory Animals and were approved by the Animal Research Committee of Keio University School of Medicine (protocol No. 12036‐8). Experiments were conducted using male and female mice aged 1–16 weeks. All mice were maintained under a 12:12 h light:dark cycle (lights on at 8:00 am). The genetic background of all transgenic and wild‐type mice was mixed C57BL/6J and 129SvEvTac. Mice were housed in standard cages with approximately five mice per cage and had ad libitum access to food and water. The weight of the mice ranged from 5 to 30 grams depending on age.

### 2.2 Generation of *LncOL1* ‐tTA knockin mice and tTA‐mediated YFP induction

We employed the CRISPR/Cas9‐mediated homologous recombination to insert a mammalianized tetracycline transactivator (tTA)‐polyA cassette downstream of the transcription start site for *LncOL1*. We selected one of seven founder lines for use as the *LncOL1*‐tTA line for the present study. To locate tTA activity, we crossed *LncOL1*‐tTA mice with Actb‐tetO‐YC‐Nano50 (Yellow Cameleon, YC; Kanemaru et al., 2014) and TIGRE‐tetO‐YCX2.60 (Madisen et al., 2015). We used these two tetO lines as yellow fluorescent protein (YFP) reporter mice (hereafter referred to as tetO‐YFP lines, because YC contains YFP), which express YC in the presence of tTA. The obtained *LncOL1*‐tTA::tetO‐YFP lines are presented as *LncOL1*‐YFP.

### 2.3 Genotyping

We used the following PCR primer sets for mouse genotyping: –212up (5′‐TGCTTCCAAACTGACATGGA‐3′) and mtTA24L (5′‐CGGAGTTGATCACCTTGGACTTGT‐3′) for *LncOL1*‐tTA mice; tetOup (5′‐AGCAGAGCTCGTTTAGTGAACCGT‐3′) and intronlow (5′‐AAGGCAGGATGATGACCAGGATGT‐3′) for tetO‐YC‐Nano50 mice; and TIGREup (5′‐GTGTAGCCCTGGCTTTTCTG‐3′) and TIGRElow (5′‐CGTAAGGCCATTATCTCTCATCC ‐3′) for tetO‐YCX2.60 mice. The sizes of the PCR products were approximately 260, 610, and 330 bp, respectively. Wild‐type mice were negative for the PCR products stated above.

### 2.4 Section preparation

The mice were deeply anesthetized with ketamine (100 mg/kg, i.p.) and xylazine (10 mg/kg, i.p.) and perfused with a 4% paraformaldehyde (PFA)/phosphate buffer (PB) solution. The brains were removed from the skull and postfixed overnight with 4% PFA/PB. The brains were cryoprotected in 20% sucrose/phosphate‐buffered saline (PBS) for 1 day and frozen. Coronal sections were cut at a thickness of 25 μm on a cryostat and mounted on silane‐coated glass slides (Matsunami Glass).

### 2.5 Immunohistochemistry

The sections were incubated with primary antibody overnight at room temperature. The following primary antibodies were used: goat anti‐GFP polyclonal antibody (1:1000 dilution, Frontier Institute, RRID:AB_2571574), rat anti‐proteolipid protein (PLP)/DM‐20 monoclonal antibody (1:20 dilution, AA3 hybridoma supernatant (Yamamura et al., 1991)), mouse anti‐CC1 monoclonal antibody (1:500 dilution, Sigma, cat No. OP80‐100UGCN), and mouse anti‐Gpr17 monoclonal antibody (1:500 dilution, Abcam, cat No. ab279382). The sections were then incubated with species‐specific secondary antibody conjugated to Alexa Fluor 488 or 594 (1:1000 dilution for each, Invitrogen, RRID:AB_2762838, AB_2535795) for 2 h at room temperature. Fluorescence images were obtained using a confocal laser scanning microscope (Fluoview FV3000, Olympus) or an inverted microscope (BZ‐X710, Keyence).

### 2.6 In situ hybridization

The sections were fixed with 4% PFA/PBS for 20 min and treated with 40 μg/mL proteinase K (Roche) at room temperature for 30 min. The sections were then postfixed with 4% PFA/PBS for 15 min to inactivate the proteinase. After acetylation with 0.25% acetic anhydride, prehybridization was conducted for 2 h at 60 °C in hybridization buffer containing 50% formamide (Wako), 50× Denhardt solution (Nacalai Tesque), and 10 mg/mL salmon sperm DNA (Invitrogen). After prehybridization, the following digoxigenin (DIG)‐labeled cRNA probes were hybridized overnight at 60 °C: *mtTA*, platelet‐derived growth factor receptor alpha (*Pdgfra*), *LncOL1*, cytidine 5′‐triphosphate synthetase (*Ctps*), carbonic anhydrase 2 (*Car2*), anillin (*Anln*), kallikrein 6 (*Klk6*), and *Plp1*. The synthesis of cRNA probes for *Pdgfra*, *LncOL1*, *mtTA*, and *Plp1* has been described previously (Ma et al., 2006; Oishi et al., 2022; Inamura et al., 2012; Kagawa et al., 1994). For the synthesis of the other probes, plasmids containing *Ctps*, *Car2*, *Anln*, and *Klk6* (NCBI accession Nos. AK148259, AK146015, AK045703, and AK138218, respectively) were used. These clones included 2726 bp of cDNA (nucleotides 53–2102, NM_016748.2), 1726 bp (nucleotides 182–1708, NM_001357334), 4599 bp (nucleotides 1014–3956, NM_028390.4), and 1115 bp (nucleotides 2–1115, NM_001164697.2), respectively, and these cDNAs were transcribed into DIG‐labeled cRNAs. After stringency washing, the sections were incubated with an alkaline phosphatase‐conjugated anti‐DIG antibody (1:5000; Roche) for 90 min at room temperature. The cRNA probes were visualized by incubation with a freshly prepared colorimetric substrate (NBT/BCIP, Roche) overnight at room temperature. After color development, the sections were counter‐stained with nuclear fast red (C.I. 60 760, Sigma). Images of the sections were captured using an inverted light microscope (BZ‐X710, Keyence).

For hybrid staining with *LncOL1* RNA and PLP/DM‐20, the colorimetric in situ hybridization (ISH) of *LncOL1* was conducted and then PLP/DM‐20 was immunolabeled with Alexa 594. For hybrid staining with RNA and YFP, the fluorescence in situ hybridization (FISH) was conducted, and then YFP was immunolabeled with Alexa 488. After hybridization with a DIG‐labeled cRNA probe, a peroxidase‐conjugated anti‐DIG antibody (Roche) was applied, and probes were visualized with Cy3 (TSA Plus Cyanine 3 System, PerkinElmer, RRID:AB_2572409).

### 2.7 Quantification of cell numbers and densities

After fluorescence images were captured, the images were analyzed, including for the cell number count, using ImageJ software (http://rsb.info.nih.gov/ij/). ImageJ was used to adjust the brightness and contrast of the images, to delineate the region of interest (ROI) and measure the area, and to count the cells using the point tool and the find maxima function.

**FIGURE 1:**
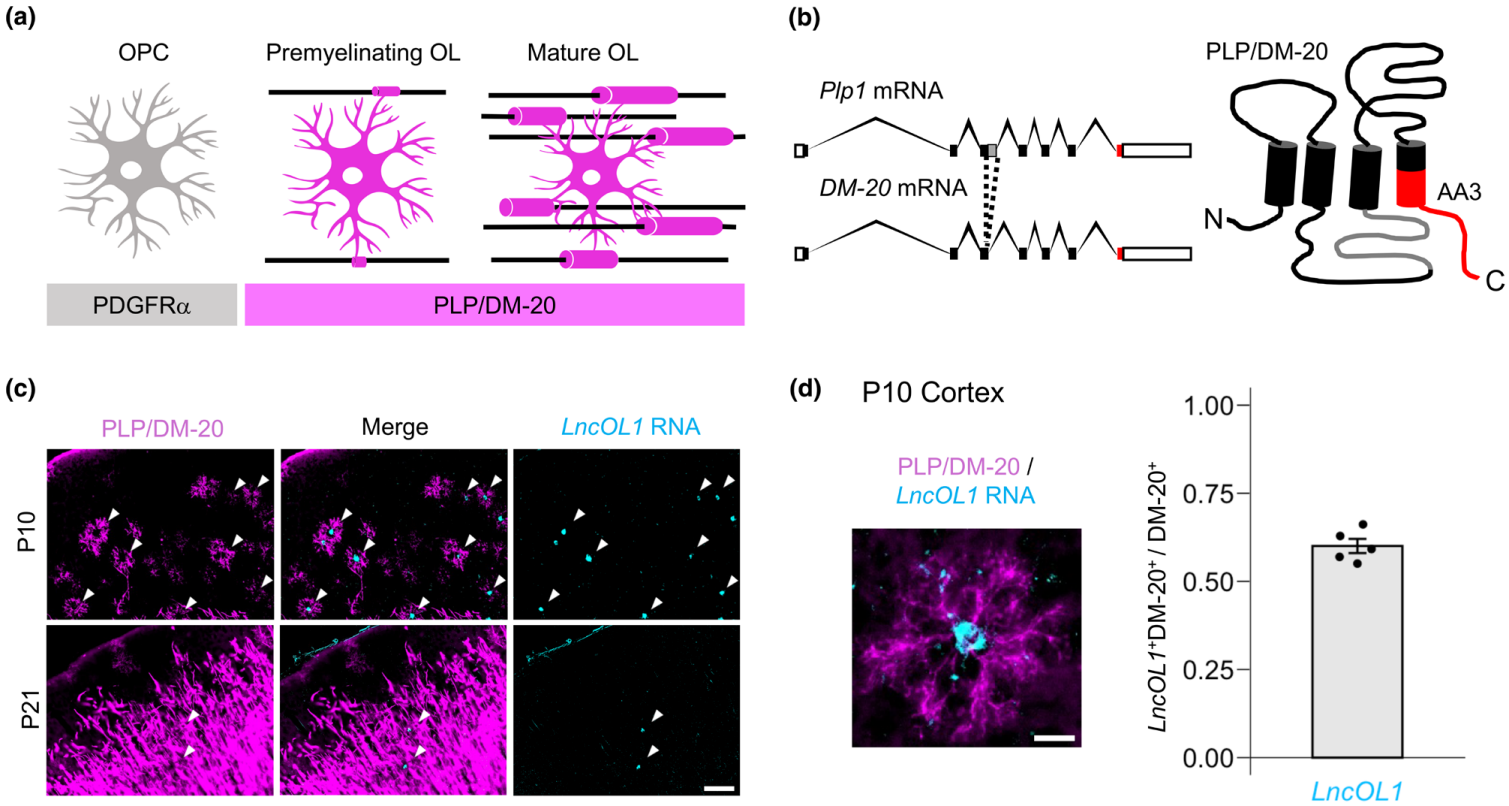
DM‐20^+^ premyelinating oligodendrocytes (OLs) express long non‐coding oligodendrocyte 1 gene (*LncOL1*) RNA. (a) Schematic illustration of OL lineage immunoreactivity. Both premyelinating OLs and mature OLs express proteolipid protein (PLP), while only premyelinating OLs express DM‐20. (b) Structures of transcripts and proteins from *Plp1*. Both *Plp1* and *DM‐20* mRNAs were of the same origin and were transcribed from *Plp1*. The third exon (gray in *DM‐20* mRNA) is spliced in *DM‐20* mRNA, and the corresponding amino acid sequence is deleted in the DM‐20 protein (gray in DM‐20 protein). Our PLP/DM‐20 antibody recognizes the amino acid sequence encoded in the shared seventh exon (red) as an antigen (AA3 epitope), thus detecting both DM‐20 and PLP. (c) DM‐20^+^ premyelinating OLs (magenta) expressed *LncOL1* RNA (cyan) in the P10 cortex. The white arrowheads indicate premyelinating OLs expressing *LncOL1* RNA. Scale bar, 100 μm. (d) A representative magnified image of a DM‐20^+^ premyelinating OL with *LncOL1* RNA expression in the P10 cortex. The bar graph shows the ratio of the number of DM‐20^+^ cells with *LncOL1* expression to the total number of DM‐20^+^ cells. *n* = 5, representing 5 mice. The bar graph is presented as the mean ± standard error of the mean. Scale bar, 20 μm.

We calculated the ratio of *LncOL1* and DM‐20 double‐positive cells to total DM‐20^+^ cells (Figure 1). The region of interest (ROI) was defined by manually delineating the cerebral cortex using the ImageJ freehand tool. For this analysis, the brightness and contrast of the acquired images were adjusted to identify DM‐20^+^ cells, and the ROI was defined to include the entire isocortex in reference to the Allen Brain Atlas (http://mouse.brain‐map.org) to consider as many cells as possible, as DM‐20^+^ cells were only sparsely distributed throughout the cortex. The sections at the region corresponding to the bregma +0.5 mm in the adult brain were used. For section preparation, the size of lateral ventricles was visually inspected to ensure that they were approximately the same size to maintain consistency between samples. Two sections were used for each individual mouse brain and the average counts of these sections were plotted.

To compare wild‐type mice and *LncOL1*‐tTA homozygotes, we quantified their PLP expression (Figures S2 and S7). For ROI delineation, the primary motor cortex was defined in reference to the Allen Brain Atlas (http://mouse.brain‐map.org). The PLP^+^ area was defined by a threshold to distinguish between the PLP^+^ and PLP^−^ areas. The ImageJ threshold function was used to generate masked images in which areas with PLP signal intensity above the threshold were converted to a mask (Figure S2). The area of the masked regions and the ROI was measured using the ImageJ measure function. Then, we estimated the ratio of PLP expression as the PLP^+^ area/ROI area (PLP^+^ + PLP^−^ areas). Three sections for each individual mouse brain were used for the analysis and the average of these sections was plotted.

To determine which OL population represents YFP‐labeled OLs, we first calculated the ratio of RNA markers expressing YFP^+^ OLs to total YFP^+^ OLs (Figure 5; Figure S6). We counted the number of YFP^+^ OLs that expressed each RNA marker per section. The number was divided by the total count of YFP^+^ cells in that section, thus representing the ratio of RNA marker and YFP double‐positive cells to YFP^+^ cells.

The RNA/YFP^+^ cell density was calculated by dividing the number of cells expressing the target RNA/YFP by the area of the ROI to analyze the expression of each RNA marker or YFP quantitatively (Figure 6; Figure S8). The bregma −0.5 mm slices were used. Each section included the cerebral cortex, the striatum, the corpus callosum, and the fimbria of the hippocampus because these areas were where YFP signals were detectable in the adult brain. The similarity of lateral ventricle and fimbria shape was used as a landmark in the preparation of sections. For analysis of ISH signals, the ROI was defined within the cerebral cortex to fit in the region approximately ranging from mediolateral 1 to 3 mm from the midline using the rectangle selection tool of the ImageJ. YFP IHC signals were sparsely observed in the adult brain, thus we took the same approach to delineate ROI as we did in counting the DM‐20^+^ cells. In Figure S8, two sections were used for each mouse and averaged values were plotted. In Figures 5, 6; Figure S6, quantification was conducted with one section for each RNA/YFP due to the limitation in the number of sections acquired from one brain sample and the amount of available reagent.

For other IHC analysis, the Gfap/Iba1 expression was presented as a ratio of the area positive for these markers to the ROI area (Figure S8). In this analysis, we used the confocal images captured with 40× objective lens. After image acquisition, we set the ROI within the deep layer of the cerebral cortex using the rectangle selection tool and created masked images to distinguish Gfap/Iba1^+^ areas versus Gfap/Iba1^−^ areas using the threshold tool of ImageJ. The area of masked regions and the ROI was measured using the ImageJ measure function. The Gfap/Iba1expression was presented as the ratio of the Gfap/Iba1^+^ area to the ROI area. Two sections for each individual mouse brain were used for the analysis and averaged values were plotted.

### 2.8 RNA extraction and qRT‐PCR of *LncOL1*

Mice were killed by cervical dislocation, and their brainstems and thalami were collected. Total RNA was isolated from the samples using TRIzol (Thermo Fisher Scientific, cat No. 15596018) and was reverse transcribed into cDNA using ReverTra Ace qPCR RT Master Mix (Toyobo, RRID:FSQ‐201). qRT‐PCR was performed using SYBR Green (Nippon Genetics, cat No. KK4605) on the Step One Plus real‐time PCR monitoring system (Thermo Fisher Scientific). The following primer sets were used (He et al., 2017): *LncOL1*‐f1, 5′‐GTACAAGACCATCCAGCATACA‐3′, *LncOL1*‐r1, 5′‐CCTTGCATGTGGCTCAATAAAG‐3′ for detecting exons I‐II; *LncOL1*‐f2, 5′‐CAGGAGATGACCACCCATTT‐3′, *LncOL1*‐r2, 5′‐ATCATGGAGCTTCAGGTTCAG‐3′ for detecting exons IV‐V; *LncOL1*‐f3, 5′‐CTGCATGCTCCACTTCAGTA‐3′, and *LncOL1*‐r3, 5′‐TATGCAAAGCCCTGGGTATC‐3′ for detecting exon VI. RNA levels were normalized to those of Gapdh and quantified using the 2^−ΔΔCT^ method.

### 2.9 X‐ray irradiation

Mice were deeply anesthetized with ketamine (100 mg/kg) and xylazine (10 mg/kg), and their heads were fixed in a stereotaxic apparatus. A lead plate was placed above the skull to protect the mouse body from X‐ray exposure. X‐ray irradiation was applied to the head through a 5 mm slit in the plate from interaural +1 mm to +6 mm using an X‐ray irradiator (AB‐160, AcroBio). A total dose of 36 Gy was fractionated into 6 Gy × 6. The heads of the mice were irradiated three times per week.

The irradiation efficiency was determined in reference to a past study (Begolly et al., 2018), where fractionation of X‐ray irradiation enhanced the loss of oligodendrocyte precursor cells (OPCs). We conducted a trial experiment to apply single dose of 20 Gy to the brain hemisphere and confirmed that the efficiency of the X‐ray irradiation was insufficient to induce the total loss of OPCs with that protocol (data not shown). We histologically determined the efficiency of the X‐ray irradiation by investigating the *Pdgfra* signals detected by ISH and chose the protocol to maximize the depletion of OPC supply.

### 2.10 Statistics

All the data are presented as the mean ± standard error of the mean. In the histological analysis, sample size, *n*, refers to the number of animals. We determined the number of biological replicates based on power analysis, with power set at 80% to detect a 20% difference between groups at a *p*‐value of 0.05. No data points were excluded and no test for outliers was performed. Normality was assessed using the Shapiro–Wilk test. Full statistical reports are provided in the Tables S1–S5. For statistical analysis of cell density via immunohistochemistry and ISH, Welch's *t* test was used for normally distributed data. Due to the limited group size (*n* = 3–4 mice per group), a few non‐normally distributed data including zero values were also analyzed by Wilcoxon rank sum test or Welch's *t* test. A two‐way repeated ANOVA was used for the qPCR analysis. Significant differences within the same primer set were then evaluated between *LncOL1*‐tTA homozygous and wild‐type mice using Welch's *t* test. Statistical processing was performed using R, version 4.2.2 (available at www.r‐project.org, RRID:SCR_001905), Excel, version 16.87 (Microsoft, RRID:SCR_016137), and MATLAB R2024a (MathWorks, RRID:SCR_001622).

## 3 RESULTS

### 3.1 *LncOL1* ^+^ NFOLs were DM‐20^+^ premyelinating OLs in the juvenile mouse brain

Premyelinating OLs have been defined as cells with DM‐20 immunoreactivity and radially extended processes (Figure 1a; Trapp et al., 1997). DM‐20 is an isoform of PLP; therefore, premyelinating OLs and myelinating OLs are both labeled with the PLP antibody against the C‐terminal peptide (Figure 1b; Yamamura et al., 1991). It was easy to separate these populations by their unique shapes when their morphologies were identifiable (post‐natal day 10, Figure 1c, top), but it was difficult to identify DM‐20^+^ premyelinating OLs at P21 because expanding PLP^+^ areas concealed them (Figure 1c, bottom).

**FIGURE 2:**
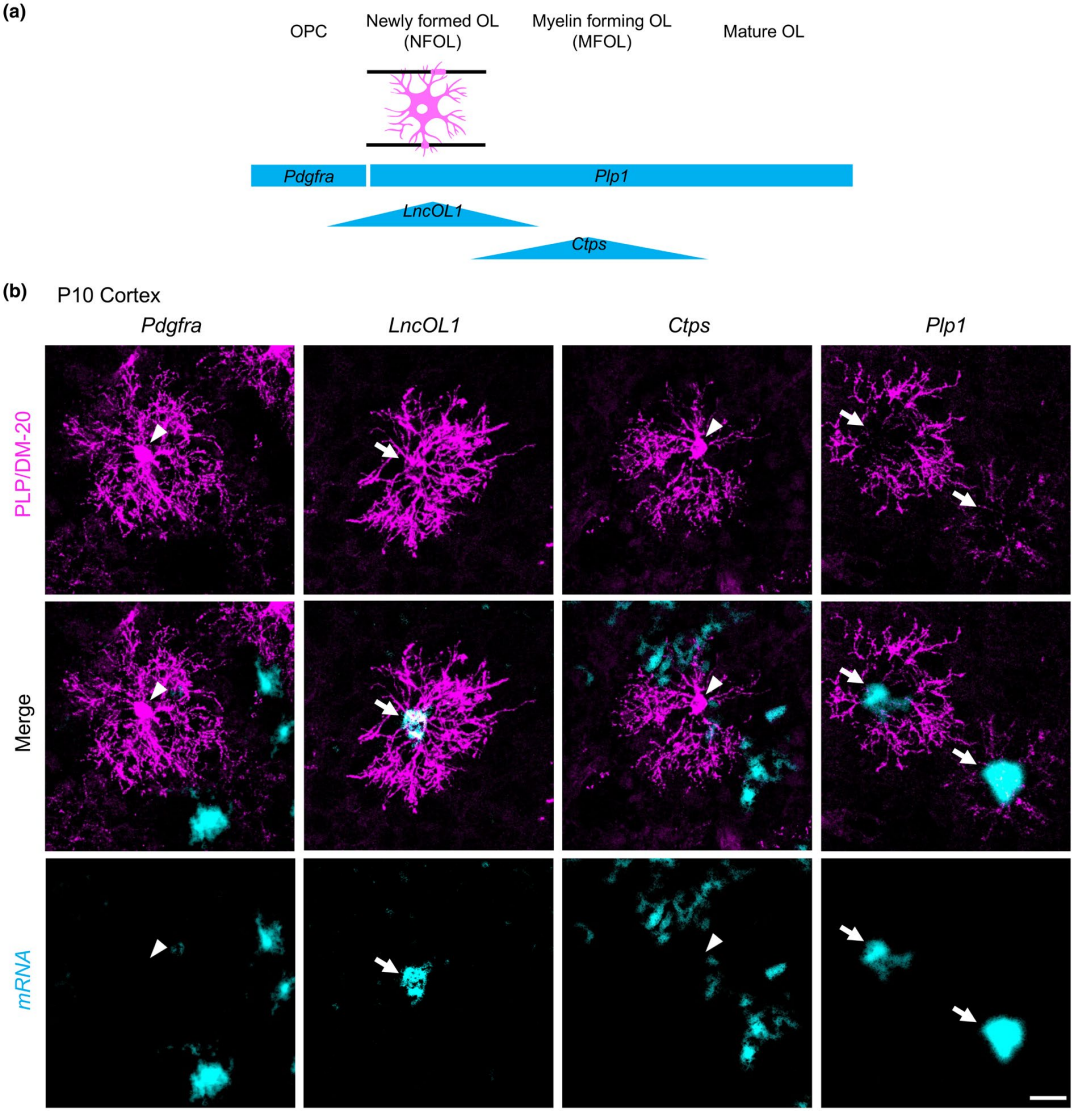
DM‐20^+^ premyelinating oligodendrocytes (OLs) correspond to newly formed oligodendrocytes (NFOLs), not to oligodendrocyte precursor cells (OPCs) and myelin‐forming oligodendrocytes (MFOLs). (a) Schematic illustration of correspondence between morphologically defined premyelinating OLs and transcriptionally defined OL populations. Marques et al. reported *Pdgfra*, *LncOL1*, and *Ctps* expression in OPCs, NFOLs, and MFOLs, respectively (Marques et al., 2016). (b) DM‐20^+^ premyelinating OLs expressed *LncOL1* and *Plp1* (arrows), but not *Pdgfra* and *Ctps* (arrowheads) in P10 mice. Scale bar, 20 μm.

Morphology has been used to segregate premyelinating OLs from myelinating OLs until recent single‐cell RNA sequencing proposed a gene expression‐based classification for OLs. Marques et al. reported that *LncOL1* is enriched in the NFOL population. We intended to bridge our current knowledge of OL gene expression with conventional immunoreactivity‐ and morphology‐dependent OL classification. To determine whether NFOLs were DM‐20^+^ premyelinating OLs, we conducted *LncOL1* ISH combined with PLP immunohistochemistry. In P10 forebrain sections, *LncOL1* RNA signals merged with those of DM‐20^+^ premyelinating OLs (Figure 1c). A total of 60.0% ± 2.0% of the DM‐20^+^ premyelinating OLs were *LncOL1* ^+^ cells (Figure 1d). DM‐20^+^ premyelinating OLs did not express *Pdgfra* (OPC marker) or *Ctps* (MFOL marker; Figure 2). The DM‐20^+^ cells were positive for Gpr17, a marker for OPCs and premyelinating OLs (Chen et al., 2009). Some Gpr17^+^ cells were positive for *LncOL1*, further supporting that premyelinating OLs expressed *LncOL1* (Figure S3). In P21 brain sections, where DM‐20^+^ premyelinating OLs were difficult to capture, we found CC1^+^ mature OLs negative for *LncOL1* RNA signals (Figure S4). Collectively, these results indicated that NFOLs and DM‐20^+^ premyelinating OLs were overlapping populations in the juvenile brain.

### 3.2 YFP‐expressing OLs in the *LncOL1* ‐YFP mouse line were a distinct population from DM‐20^+^ premyelinating OLs

**FIGURE 3:**
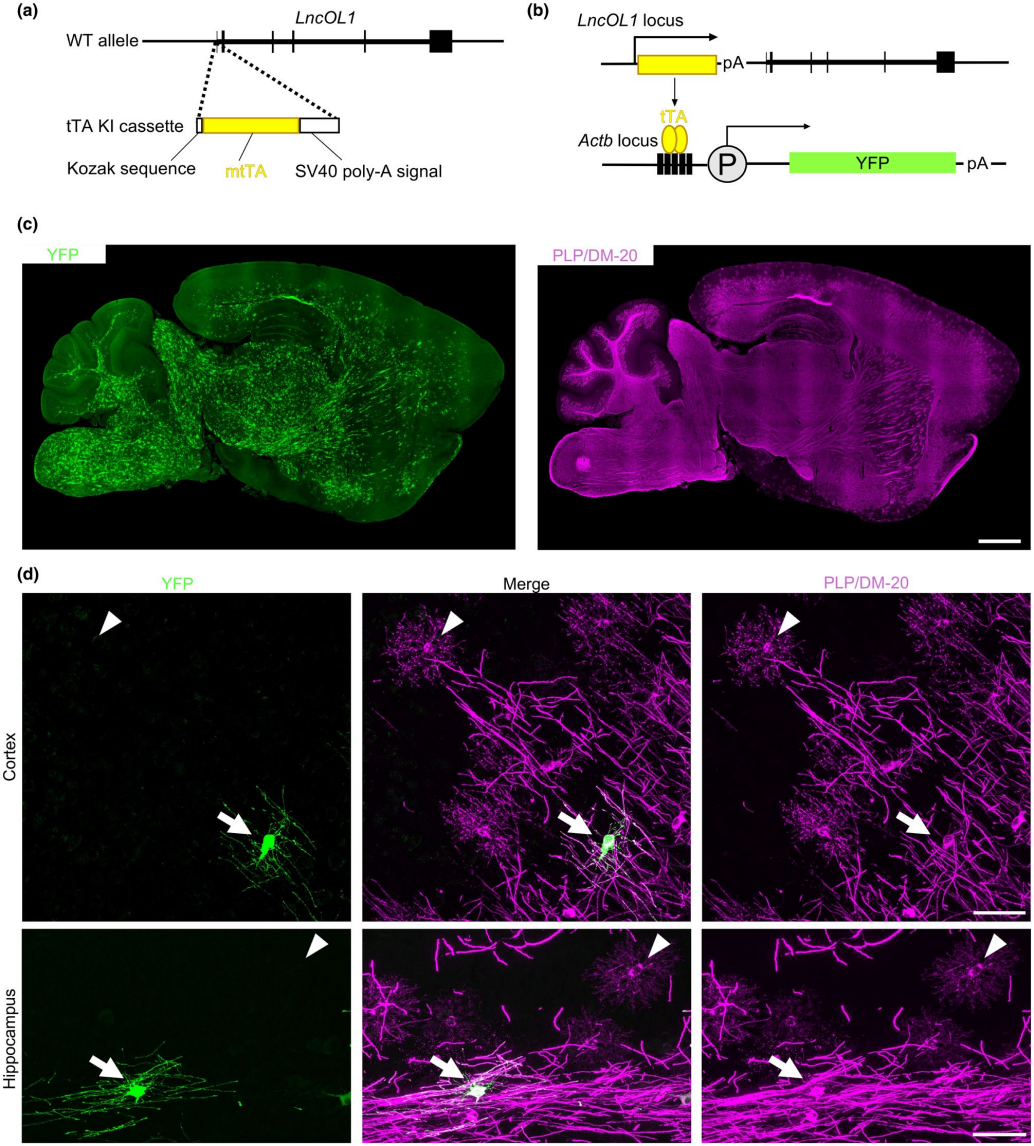
Establishing the *LncOL1*‐YFP mice. (a) Generation of *LncOL1*‐tTA mice. The tetracycline transactivator (tTA)‐coding sequence was inserted with the Kozak sequence and SV40 polyadenylation site into the wild‐type *LncOL1* locus by CRISPR. (b) Yellow fluorescent protein (YFP)‐induction using a tet‐system. *LncOL1*‐tTA mice were crossed with tetO‐YFP mice. We then obtained double‐transgenic mice, harboring *LncOL1*‐tTA::TetO‐YFP (*LncOL1*‐YFP). (c) Representative images of *LncOL1* RNA or YFP induced in the developing *LncOL1*‐YFP mouse brain. Scale bar, 1 mm. (d) Representative images of YFP‐labeled oligodendrocytes (OLs) in the cortex (upper panels) and the hippocampus (lower panels) of P16 *LncOL1*‐YFP mice. Scale bars, 50 μm.

To overcome the limitation in the identification of DM‐20^+^ premyelinating OLs, we attempted to visualize premyelinating OLs by the predominant *LncOL1* expression in this population. We inserted an *mtTA*‐polyA cassette downstream of the *LncOL1* transcription start site (Figure 3a). Although it was not clear whether the *mtTA* embedded in non‐coding RNA was translated into tTA protein, tTA‐mediated YFP induction was observed in combination with different tetO‐YFP lines (Figure 3b,c). These data indicated that functional tTA protein was expressed in the *LncOL1‐*tTA mice.

**FIGURE 4:**
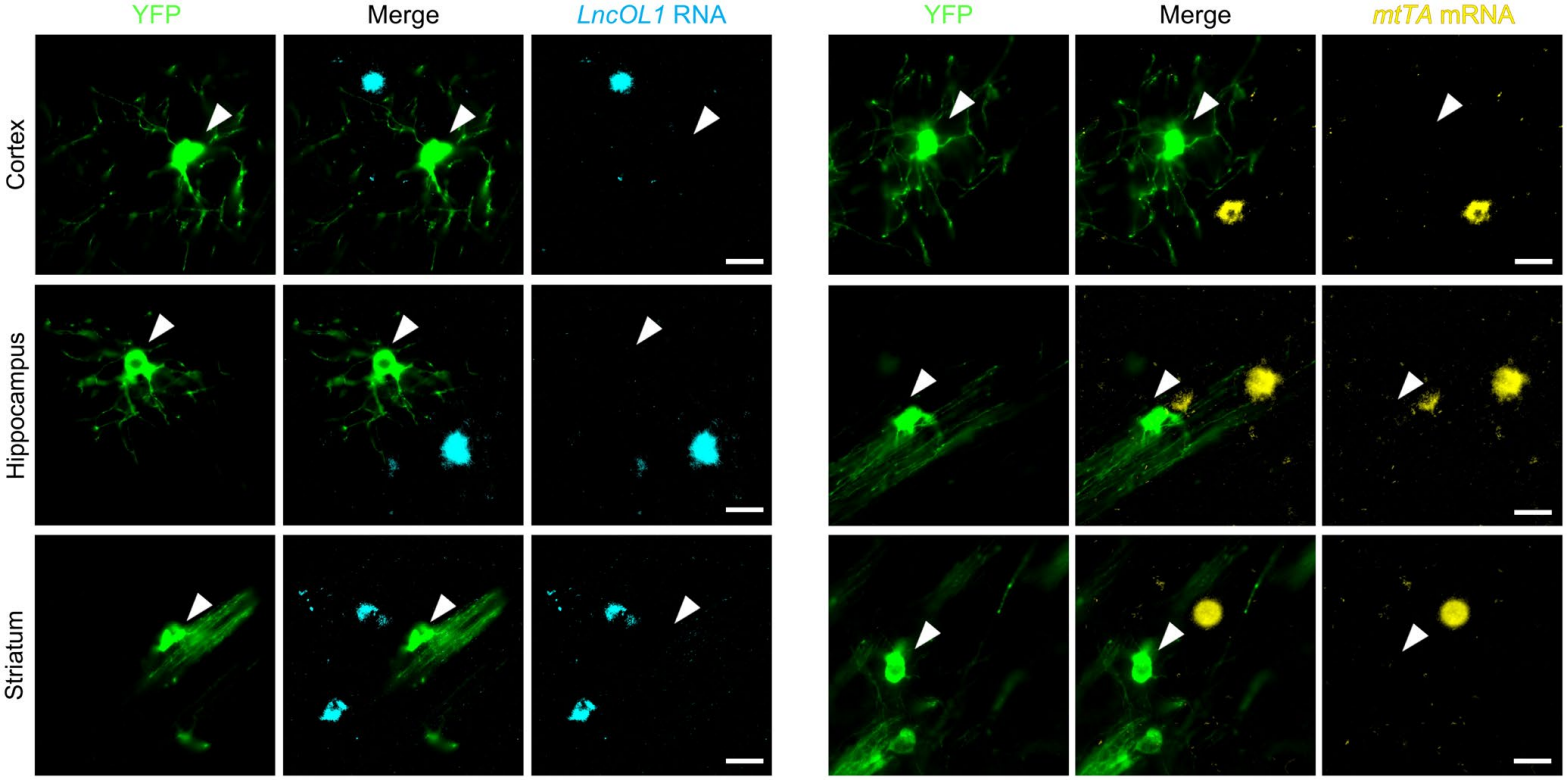
YFP^+^ cells did not express the *LncOL1* (left panels) or *mtTA* mRNA (right panels) in the P16 *LncOL1*‐YFP mouse brain. Scale bars, 20 μm.

Because we used the *LncOL1* promoter to drive tTA, YFP^+^ OLs were expected to be *LncOL1* ^+^ NFOLs or an overlapping population of DM‐20^+^ premyelinating OLs. The immunohistochemical findings demonstrated that YFP^+^ OLs were morphologically distinct from premyelinating OLs with radial processes (Figure 3d). They rather possessed PLP^+^ parallel processes (Figure 3d and Figure S5). These YFP^+^ OLs expressed neither *LncOL1* nor *mtTA* (Figure 4), suggesting that the span of *LncOL1* or *mtTA* expression was short, and tTA‐mediated YFP expression likely occurred in the next differentiated population, MFOLs.

### 3.3 YFP ^+^ OLs were MFOLs in *LncOL1* ‐YFP mice

**FIGURE 5:**
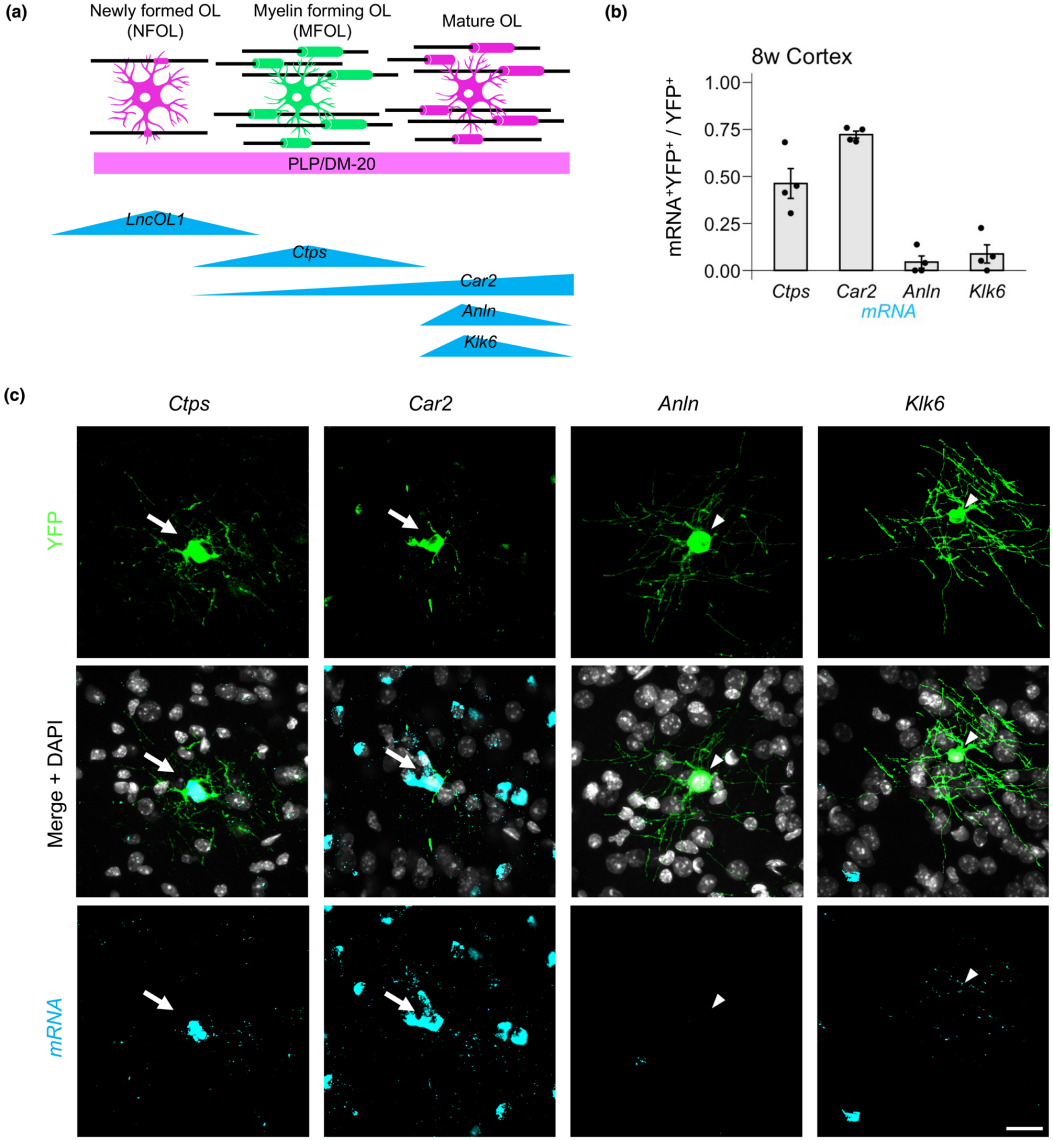
Visualized oligodendrocytes (OLs) in the adult *LncOL1*‐YFP mice are myelin‐forming oligodendrocytes (MFOLs). (a) Schematic illustration of RNA expression in yellow fluorescent protein (YFP)‐labeled cells (green). Markers with high expression and specificity in a given OL population (newly formed oligodendrocytes (NFOLs), MFOLs, and mature oligodendrocytes (MOLs)) were chosen from the single‐cell RNA seq data (Marques et al., 2016). The in situ hybridization signal distribution was checked in the Allen ISH database (https://mouse.brain‐map.org), and RNA expression was confirmed in the Allen Single‐cell RNA SMART‐seq database (https://portal.brain‐map.org/atlases‐and‐data/rnaseq) and the Allen Brain Cell database (https://knowledge.brain‐map.org). (b) RNA marker expression of YFP^+^ cells in the cortex of post‐natal week 8 *LncOL1*‐YFP mice. The bar graph shows the ratio of the number of RNA marker‐positive cells with YFP expression to the total number of YFP^+^ cells. (*n* = 4 mice). The data are presented as the mean ± standard error of the mean. (c) Representative images of YFP‐labeled OLs expressing the MFOL RNA markers, *Ctps* and *Car2*, but not the MOL markers, *Anln* and *Klk6* in the cortex of post‐natal week 8 *LncOL1*‐YFP mice. The white staining is DAPI. Scale bar, 20 μm.

We next investigated which OL population in the mice was labeled with YFP. We selected the following population‐specific mRNAs: *Ctps* (MFOL marker; Marques et al., 2016), *Car2* (MFOL and MOL marker; Marques et al., 2016; Mei et al., 2021), *Anln* (MOL marker; Erwig et al., 2019; Marques et al., 2016; Patzig et al., 2016), and *Klk6* (MOL marker; Marques et al., 2016; Terayama et al., 2004; Yamanaka et al., 1999; Figure 5a) and conducted colorimetric ISH. We confirmed that the ISH signals obtained from our probes were consistent with those seen in the Allen Brain Atlas ISH database (https://mouse.brain‐map.org). We performed FISH for the respective gene followed by YFP immunohistochemistry in adult mouse brains (>post‐natal week 8; Figure 5b,c). We found that 46.2% ± 7.9% of the YFP‐expressing cells were *Ctps* ^+^ (MFOLs), 72.2% ± 1.9% were *Car2* ^+^ (MFOLs and MOLs), 4.4% ± 3.3% were *Anln* ^+^ (MOLs), and 8.8% ± 4.9% were *Klk6* ^+^ (MOLs; Figure 5b). In young mice (post‐natal week 4), stage marker expression profiles within YFP^+^ cells resembled those in adult mice (Figure S6). These data indicated that most YFP^+^ OLs belong to MFOLs and are a younger population among myelinating OLs.

### 3.4 Knockdown of *LncOL1* RNA did not reduce the immunoreactivity for PLP in the developing brain

We considered two potential uses of the established *LncOL1*‐tTA mouse: (1) tTA‐mediated transgene induction by crossing tetO lines and (2) *LncOL1* RNA knockdown by generating homozygotes. In the *LncOL1*‐tTA mice, the SV40 polyadenylation signal follows tTA cDNA; therefore, the transcription under the control of the *LncOL1* promoter should stop at the artificial polyadenylation signal, and downstream *LncOL1* RNA expression should be suppressed. If an inserted polyadenylation signal was effective, *LncOL1* RNA would be depleted in *LncOL1*‐tTA homozygotes (Figure S7a). We examined whether homozygous *LncOL1*‐tTA mice phenocopied the *LncOL1*‐KO mice, in which a reduced number of mature OLs and thinner myelin sheaths were observed in the developing mouse brain (He et al., 2017). First, we performed RT‐qPCR to measure the expression level of *LncOL1* RNA (Figure S7a). Unexpectedly, *LncOL1*‐tTA homozygotes retained 15%–50% of *LncOL1* RNA (Figure S7b). We then examined the PLP immunoreactivity in the developing cerebral cortex of wild‐type and homozygous *LncOL1*‐tTA mice but did not detect a difference (Figure S7c). These data suggest that a fraction of the *LncOL1* RNA sequence remains in *LncOL1*‐tTA homozygotes, such that knockdown of *LncOL1* RNA did not result in dysmyelination.

### 3.5 Adult OPCs proliferate and differentiate to form NFOLs and MFOLs

**FIGURE 6:**
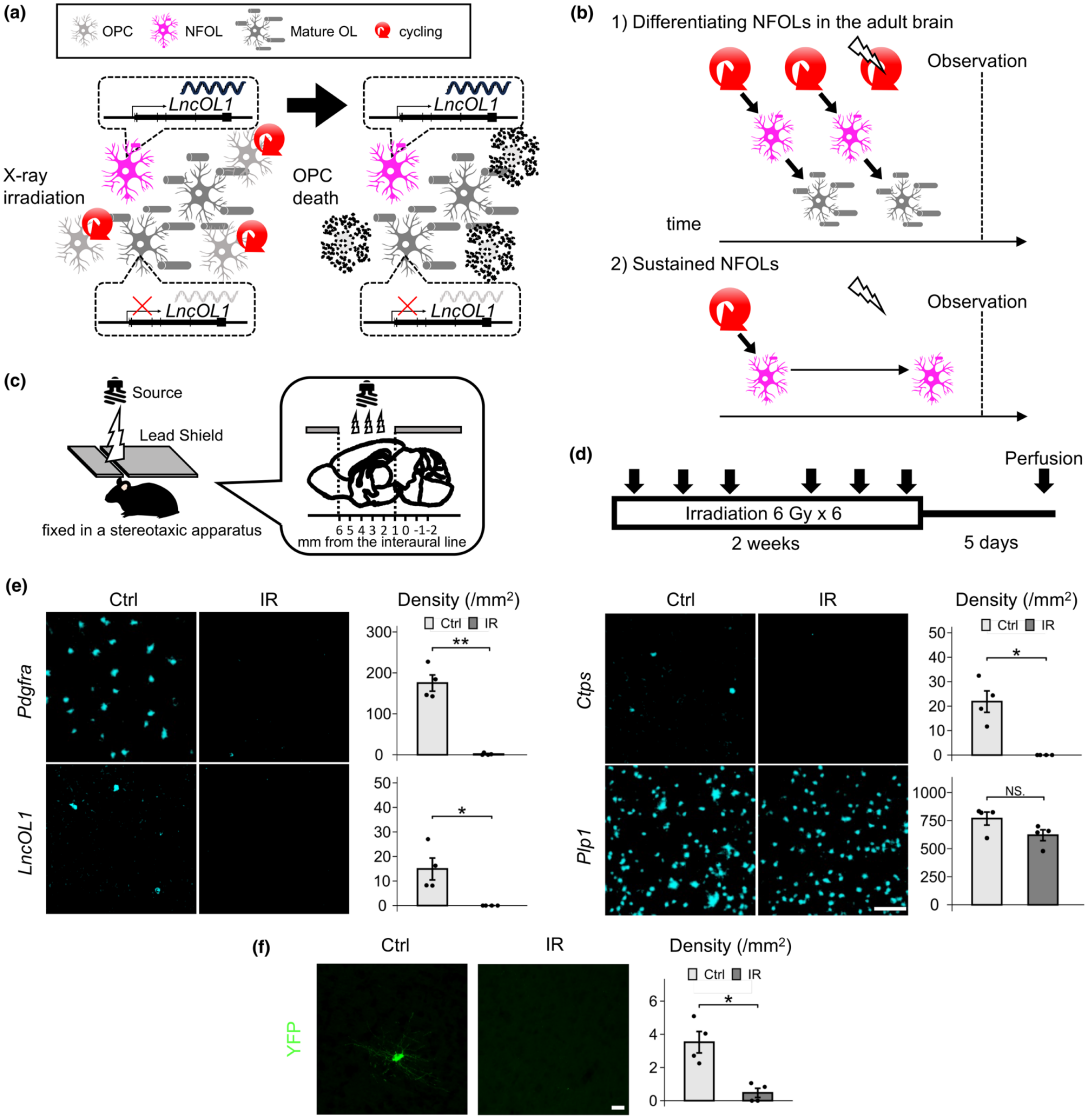
Adult brain oligodendrocyte precursor cells (OPCs) proliferate and continuously differentiate. (a, b) In adult brains, if constant replenishment of newly formed oligodendrocytes (NFOLs) is achieved by OPC proliferation and NFOLs actively differentiate, X‐ray irradiation will lead to decreased NFOLs (1). Alternatively, if NFOLs were formed in the developing brain and arrest their differentiation status, X‐ray irradiation will not reduce the number of NFOLs (2). (c) Schematic illustration of X‐ray irradiation experiments in which OPCs were depleted. The head of a post‐natal week 9–10 *LncOL1*‐YFP mouse was fixed in a stereotaxic apparatus. The mouse body was shielded from X‐rays by a lead plate. Cranial X‐ray irradiation was applied through a 5 mm slit in the plate ranging from interaural +1 mm to +6 mm. (d) Timeframe of the protocol. A total of 36 Gy irradiation was fractionated into 6 Gy per dose ×6 in 2 weeks. Surviving mice were perfused 5 days after the final irradiation. (e) In situ hybridization (ISH) signal density was compared between non‐irradiated and irradiated brains. *Pdgfra* ^+^ OPCs, *LncOL1* ^+^ NFOLs, and *Ctps* ^+^ myelin‐forming oligodendrocytes (MFOLs) were not detected in the irradiated brain. The density of *Plp1* ^+^ OLs decreased slightly, but the difference was not significant (NS). Scale bar, 100 μm. (f) The number of yellow fluorescent protein (YFP)^+^ cells also decreased after X‐ray irradiation. Scale bar, 20 μm. For comparisons between two groups, two‐tailed unpaired *t* tests were performed. *p* < 0.05 was considered the threshold for significance. The results are presented as *p < 0.05, **p < 0.01, ***p < 0.001, and NS. *n* = 4 independent brains for both the irradiated and non‐irradiated groups. The bar graphs are presented as the mean ± standard error of the mean.

Although *LncOL1* RNA expression is known to be down‐regulated in the adult brain (He et al., 2017; Kasuga et al., 2019; Yamazaki et al., 2021), *LncOL1* RNA in wild‐type mice and YFP in *LncOL1*‐YFP mice were still detectable in the adult brain. We assumed that OPCs continue to proliferate and actively differentiate into MOLs in the adult brain, and that intermediate populations, NFOLs and MFOLs, are present as a consequence of adult oligodendrogenesis (Figure 6a,b). Alternatively, OPCs in the developing brain produced NFOLs and MFOLs, and these populations sustained their differentiation stage without full maturation in the adult brain (Figure 6b).

To discern the abovementioned situations, we depleted proliferating OPCs by X‐ray irradiation. *LncOL1*‐YFP mice were exposed to 6 Gy irradiation ×6 (total 36 Gy) within 2 weeks and perfused 5 days after the final irradiation (Figure 6c,d; Begolly et al., 2018). In addition to depleting *Pdgfra* ^+^ OPCs (Figure 6e), *LncOL1* ^+^ NFOLs or *Ctps* ^+^ MFOLs were also depleted (Figure 6e). The density of *Plp1* ^+^ OLs tended to be decreased slightly compared with that of the control, but the difference was not significant (Figure 6e). The YFP^+^ OLs were also depleted by the X‐ray irradiation in the *LncOL1*‐YFP mice (Figure 6f).

As previously reported, microglia and astrocytes are activated after X‐ray irradiation and glial activation may damage the intermediate populations (Hwang et al., 2006). We investigated glial activation 24 hours after single dose of 6 Gy X‐ray irradiation, but we did not detect apparent glial activation (Figure S8). We did not detect decreased number of NFOLs and MFOLs (Figure S8), suggesting the less contribution of glial activation in cell loss of NFOLs and MFOLs. Collectively, these findings support active oligodendrogenesis in the adult brain and suggest that NFOLs and MFOLs are intermediate and transient populations in this process.

## 4 DISCUSSION

Changes in OL morphology and gene expression during differentiation have been independently described (Berry et al., 2002; Chittajallu et al., 2004; Fulton et al., 1992; Jäkel et al., 2019; Kukley et al., 2007, 2008; Marques et al., 2016; Spitzer et al., 2019; Toth et al., 2021; Trapp et al., 1997; Zeisel et al., 2015). Here, we demonstrated that morphologically defined premyelinating OLs correspond to genetically defined NFOLs and that myelinating OLs correspond to MFOLs and MOLs. Among the myelinating OLs, both younger MFOLs and older MOLs exist in the adult brain. Furthermore, as suggested by our X‐ray irradiation experiment, NFOLs and MFOLs are not quiescent but rather intermediate in the transition to MOLs. We also inferred from our results that NFOLs and MFOLs are consecutive populations; in the *LncOL1*‐YFP mouse, we expected to label NFOLs, which represent an overlapping population with premyelinating OLs, but YFP‐labeled OLs were morphologically myelinating OLs and transpired to be MFOLs.

The delayed YFP expression in MFOLs led us to speculate on the timeframe of the appearance of NFOLs in the *LncOL1*‐YFP mouse. This has not been addressed by single‐cell RNA sequencing data because they capture snapshots of gene expression at the time of sample collection. In the *LncOL1*‐YFP mouse, YFP expression requires two steps: (1) tTA production and (2) tTA‐mediated YFP induction. For the first step, considering the example of general transfection experiments in vitro (Jiang & Chen, 2006), we assumed that the translation of tTA proteins would be completed within a few hours after *mtTA* RNA transcription. The second step should take a maximum of 20 h from the tTA binding to the tetO sequence to finally drive YFPs (Gossen et al., 1995). However, the latest version, Tet‐on 3G, shortened the induction time to 30 min (Fan et al., 2012). We used the tTA gene cassette with the same construction as the tTA‐Advanced cassette used by Urlinger et al. (2000), which is one generation older than the version of Tet‐on 3G, so the time required for protein induction would be somewhere between 30 min and 20 h. Collectively, we estimated the YFP expression lag time to range from 4 to 24 h (Figure S9), and the timeframe of NFOLs in the adult brain would be less than that. This is consistent with the observation of OPCs differentiating into myelinating OLs in well‐known experimental systems in vitro, where the number of A2B5^+^ cells that were galactocerebroside^+^ in serum‐free cultures increased dramatically from 18 h after plating (Raff et al., 1983).

To confirm these estimates, we can propose in vivo imaging experiments to directly measure the duration during which NFOLs exist in the adult brain (Figure S9). For example, crossing the *LncOL1*‐YFP line with the NG2DsRedBAC transgenic line (Zhu et al., 2008) and imaging the obtained mouse brain will capture every moment of DsRed disappearance and YFP appearance. The time between these events will differentiate the time for which OLs exist as NFOLs, and we can estimate that the optimal imaging interval is from 4 to 24 h. However, this approach has some technical limitations because the fluorescence intensity in *LncOL1*‐YFP mice needs to be optimized. In addition, the distribution of the YFP‐labeled OLs is so sparse that it should be difficult to capture a sufficient number of cells in a single field of view, making it challenging to capture the initiation of ensheathment directly.

Our X‐ray irradiation results supported the idea that YFP‐labeled OLs differentiated from OPCs in the adult brain. X‐ray irradiation depleted OPCs, the source of adult oligodendrogenesis, which led to the subsequent loss of YFP^+^ OLs. We cannot completely deny the direct effects of X‐rays on the YFPs and MFOLs. In other words, their disappearance after X‐ray irradiation might not be a consequence of OPC depletion. X‐ray irradiation damages DNA (Ono & Okada, 1974) and decreases some of the expression of some mRNAs (Kerr et al., 1998), so it is possible that decreases in the number of ISH or immunoreactivity signals were due to non‐OPC‐specific depletion. By contrast, the overall number of *Plp1* signals was not as strongly affected as the number of *Pdgfra* signals, suggesting that OPCs are more vulnerable to X‐ray irradiation than the rest of the OL‐lineage population. Combined with the finding that demyelination after X‐ray irradiation likely occurs in a chronic timeframe (Panagiotakos et al., 2007), which was not the case in our experiment, we concluded that the disappearance of *LncOL1*, *Ctps*, and YFP signals was triggered by the acute loss of OPCs, which normally supply NFOLs and MFOLs via adult oligodendrogenesis.

We chose an *LncOL1* promoter to drive tTA. One question is whether functional mRNAs can be produced under the control of a promoter, which originally does not initiate the transcription of a protein‐coding gene. In general, whether protein‐coding genomic sequences produce functional proteins under the control of a long non‐coding RNA promoter remains elusive. In such long non‐coding RNAs, as seen for metastasis‐associated lung adenocarcinoma transcript 1 (*MALAT1*), protein‐coding transcripts derived from a knockin sequence downstream of the endogenous long non‐coding RNA (*MALAT1*) promoter were properly 5′‐m^7^Capped and translated into proteins (Gutschner et al., 2011). However, if post‐transcriptional modifications unique to protein‐coding gene promoters were needed, the exogenous knockin cassette downstream of the long non‐coding RNA promoter would lose the capacity to induce functional proteins. In our generated *LncOL1*‐tTA mice, *mtTA* RNAs were detected by ISH and sufficient expression of the tTA protein was confirmed by crossing the *LncOL1*‐tTA line with the tetO‐YFP reporter line, which indicated that the post‐transcriptional processing of the *mtTA* RNA proceeded without major obstacles.

A major advantage of our approach is that crossing the *LncOL1*‐tTA line with other tetO reporter lines will expand the repertoire of available tools. In the present study, we used tetO‐YC‐Nano50 (Kanemaru et al., 2014) and tetO‐YCX2.60 (Madisen et al., 2015) as reporter lines, which can also be applied to intracellular calcium monitoring. Other variations of the tetO‐GCaMP lines are used in calcium imaging (Tanaka et al., 2021). Optogenetic intervention in the target cell population is also possible by crossing the line with others, including tetO‐photoadenylylcyclase (Abe et al., 2021), tetO‐channelrhodopsin (Tanaka et al., 2012), and tetO‐archaerhodopsin (Tsunematsu et al., 2013).

Our present study supports the hypothesis that myelinating OLs include heterogeneous populations in terms of gene expression (Marques et al., 2016). Yamazaki et al. (2021) demonstrated that younger myelinating OLs with high expression of the Na^+^‐K^+^‐Cl^−^ cotransporter 1 (*Nkcc1*) contribute more to neuronal plasticity than older myelinating OLs with low expression of *Nkcc1*. They did not state that younger myelinating OLs correspond to MFOLs and that older myelinating OLs correspond to MOLs. Nevertheless, challenging the cell population heterogeneity identified according to their gene expression profiles and reconciling cellular function and gene expression with new classifications is warranted. The integration of transcriptional properties, morphology, and function in OL lineage cells is a key to understanding their heterogeneity. In this step, the visualization of a specific population can provide insight into the diverse physiology of OLs.

## AUTHOR CONTRIBUTIONS

**Kiichi Yokoyama:** Conceptualization; funding acquisition; investigation; writing – original draft; writing – review and editing; formal analysis. **Yuichi Hiraoka:** Conceptualization; resources. **Yoshifumi Abe:** Conceptualization; writing – review and editing. **Kenji F. Tanaka:** Conceptualization; funding acquisition; project administration; resources; supervision; writing – review and editing.

## CONFLICT OF INTEREST STATEMENT

The authors declare that the research was conducted in the absence of any commercial or financial relationships that could be construed as potential conflicts of interest.

### PEER REVIEW

The peer review history for this article is available at https://www.webofscience.com/api/gateway/wos/peer‐review/10.1111/jnc.16218.

## Supporting information


Figure S1.
Figure S2.
Figure S3.
Figure S4.
Figure S5.
Figure S6.
Figure S7.
Figure S8.
Figure S9.
Table S1.
Table S2.
Table S3.
Table S4.
Table S5.

## Data Availability

The data that support the findings of this study are available from the corresponding author upon reasonable request.
