# Supplementary material for: Visualization of myelin‐forming oligodendrocytes in the adult mouse brain
Source: J Neurochem. 2024 Sep 4;169(1):e16218. doi: 10.1111/jnc.16218 (PMC11657928; doi:10.1111/jnc.16218)
Supplement: Supplementary file 1 — Figure S1. Figure S2. Figure S3. Figure S4. Figure S5. Figure S6. Figure S7. Figure S8. Figure S9. Table S1. Table S2. Table S3. Table S4. Table S5. [file JNC-169-0-s001.pdf]

Supplementary Material

Title:

Visualization of myelin-forming oligodendrocytes in the adult mouse brain

Authors:

Kiichi Yokoyama[1], Yuichi Hiraoka[2,3], Yoshifumi Abe[1], and Kenji F. Tanaka[1*]

1) Division of Brain Sciences, Institute for Advanced Medical Research, Keio University School of Medicine, Tokyo, Japan.

2) Laboratory of Molecular Neuroscience, Medical Research Institute, Tokyo Medical and Dental University, Tokyo, Japan.

3) Laboratory of Genome Editing for Biomedical Research, Medical Research Institute, Tokyo Medical and Dental University, Tokyo, Japan.

*Correspondence:

Kenji F. Tanaka, Ph.D., Division of Brain Sciences, Institute for Advanced Medical Research, Keio University School of Medicine, 35 Shinanomachi, Shinjuku, Tokyo 160-8582, Japan.

Email: kftanaka@keio.jp

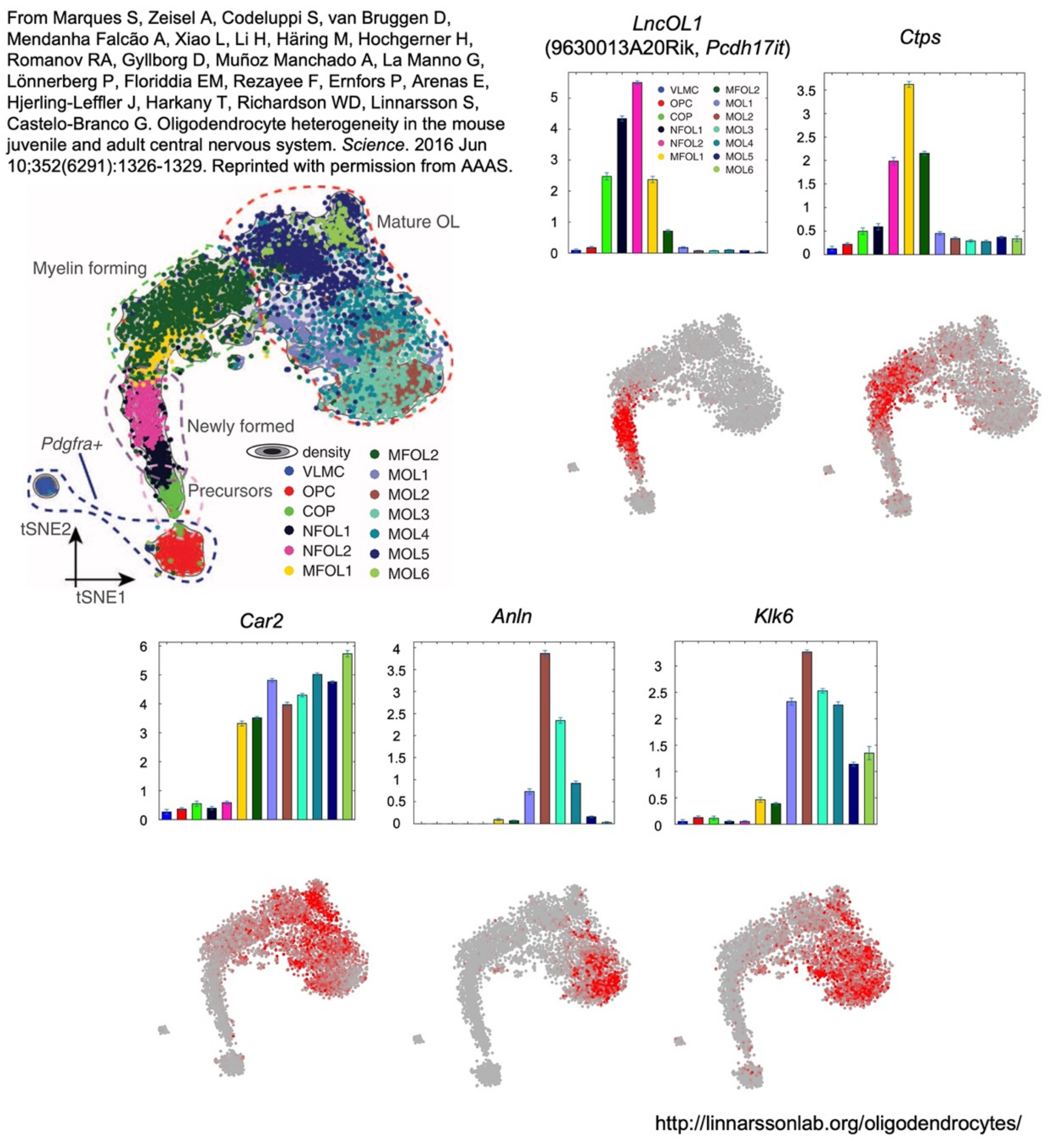

FIGURE S1 Single-cell RNA sequencing results from Marques et al. (2016). The five RNA markers used in our study are listed. In these images, for example, a population named newly formed oligodendrocyte (NFOL) (black, magenta) showed high long noncoding oligodendrocyte 1 gene (*LncOL1*) expression, and myelin-forming oligodendrocyte (MFOL1) (yellow) had high expression of *Ctps*. Other markers, such as *Car2*, *Anln*, and *Klk6* were enriched in mature oligodendrocytes (MOLs). tSNE-map with annotation is from Figure 2a, Marques et al. (2016) and other figures are created from http://linnarssonlab.org/oligodendrocytes/.

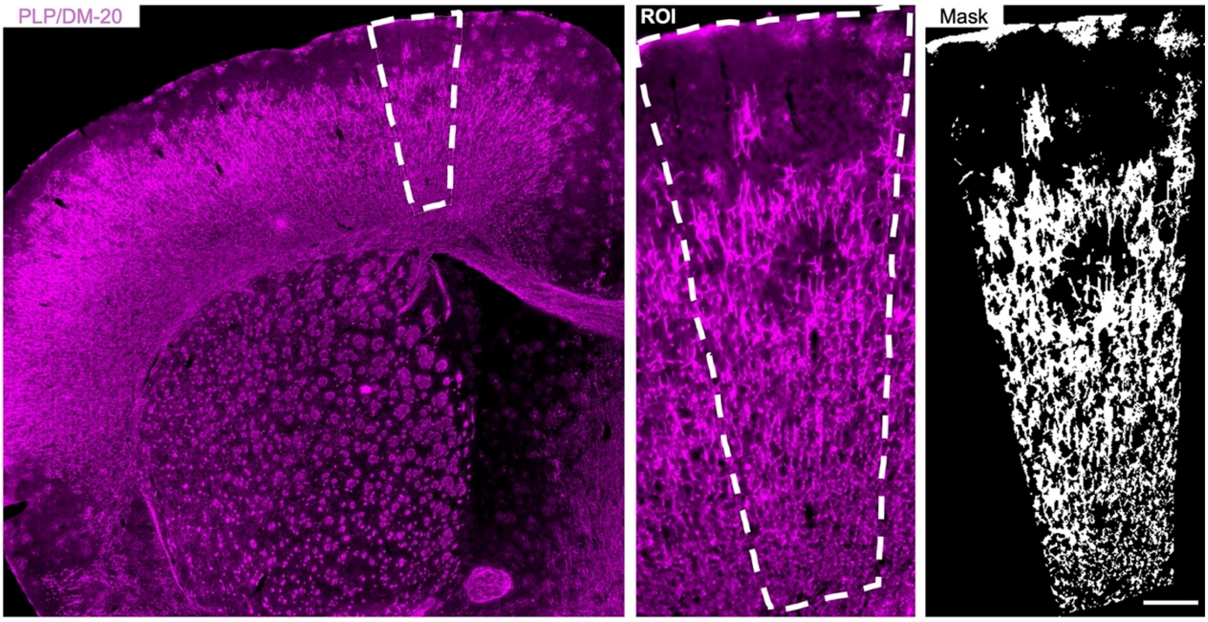

FIGURE S2 Representative images for the region of interest (ROI) delineation in the PLP expression analysis. The ROI was defined as regions including the primary motor cortex in reference to the Allen Brain Atlas (http://mouse.brain-map.org). Areas with PLP expression above threshold were converted into the mask. Scale bar, 100 μm.

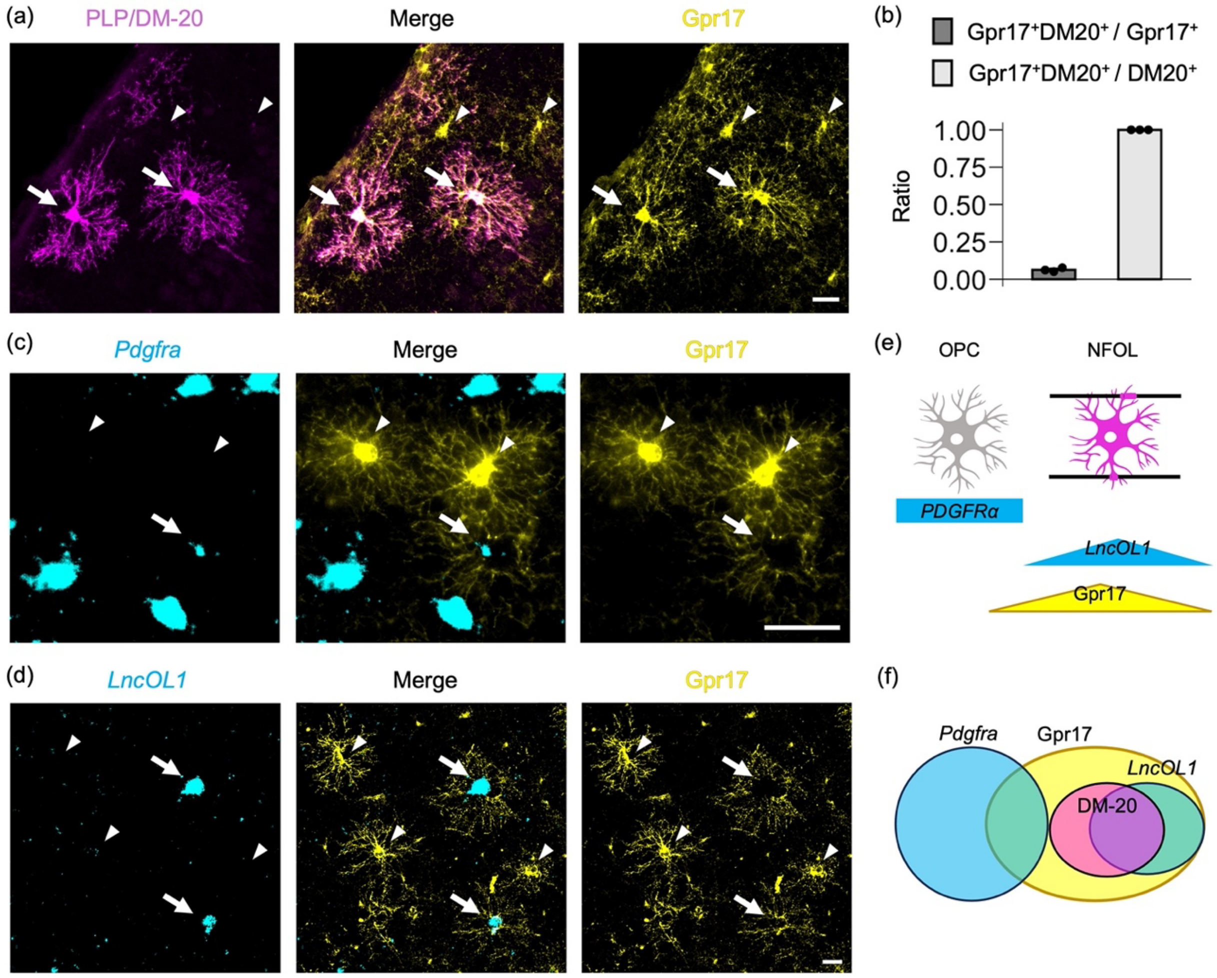

FIGURE S3 *LncOL1* expression in premyelinating oligodendrocytes (OLs) in the P10 cerebral cortex. (a) DM-20$^+$ premyelinating OLs expressed Gpr17 (Left). A large proportion of Gpr17$^+$ cells presented high immunoreactivity for Gpr17 in soma (arrowheads), while others with more elaborate processes tended to express DM-20 (arrows). Scale bar, 20 μm. Gpr17$^+$ OLs encompassed DM-20$^+$ premyelinating OLs (right, *n* = 3 mice). When measured within a single field of view using 40x objective lens of confocal microscope, 6.3% ± 0.7% of Gpr17$^+$ cells expressed DM-20 with obvious premyelinating morphology. The data are presented as the mean ± standard error of the mean. (b) Gpr17$^+$ premyelinating OLs were the overlapping population of *Pdgfra*$^+$ oligodendrocyte precursor cells (OPCs). Scale bar, 20 μm. (c) *LncOL1* is expressed in Gpr17$^+$ OLs. Scale bar, 20 μm. (d) Schematic illustration of *LncOL1* expression in premyelinating OLs. (e) *LncOL1* RNA signal colocalized with DM-20 and Gpr17 immunohistochemical signals.

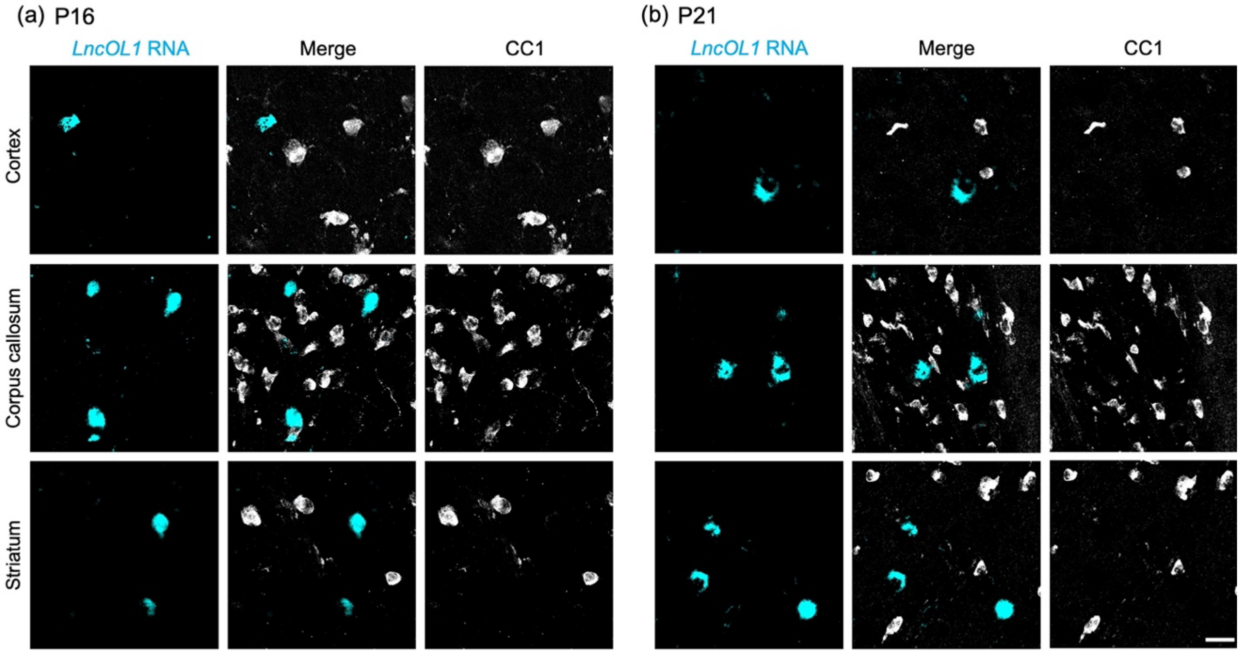

FIGURE S4 Presence of *LncOL1*$^-$ mature oligodendrocytes (OLs). (a) CC1$^+$ mature OLs did not express *LncOL1* in the cerebral cortex, the corpus callosum, and the striatum in P16 mouse brains. (b) In the P21 brains, where DM-20$^+$ premyelinating OLs were not captured due to the presence of dense myelin, CC1$^+$ mature OLs did not express *LncOL1.* Scale bar, 20 μm.

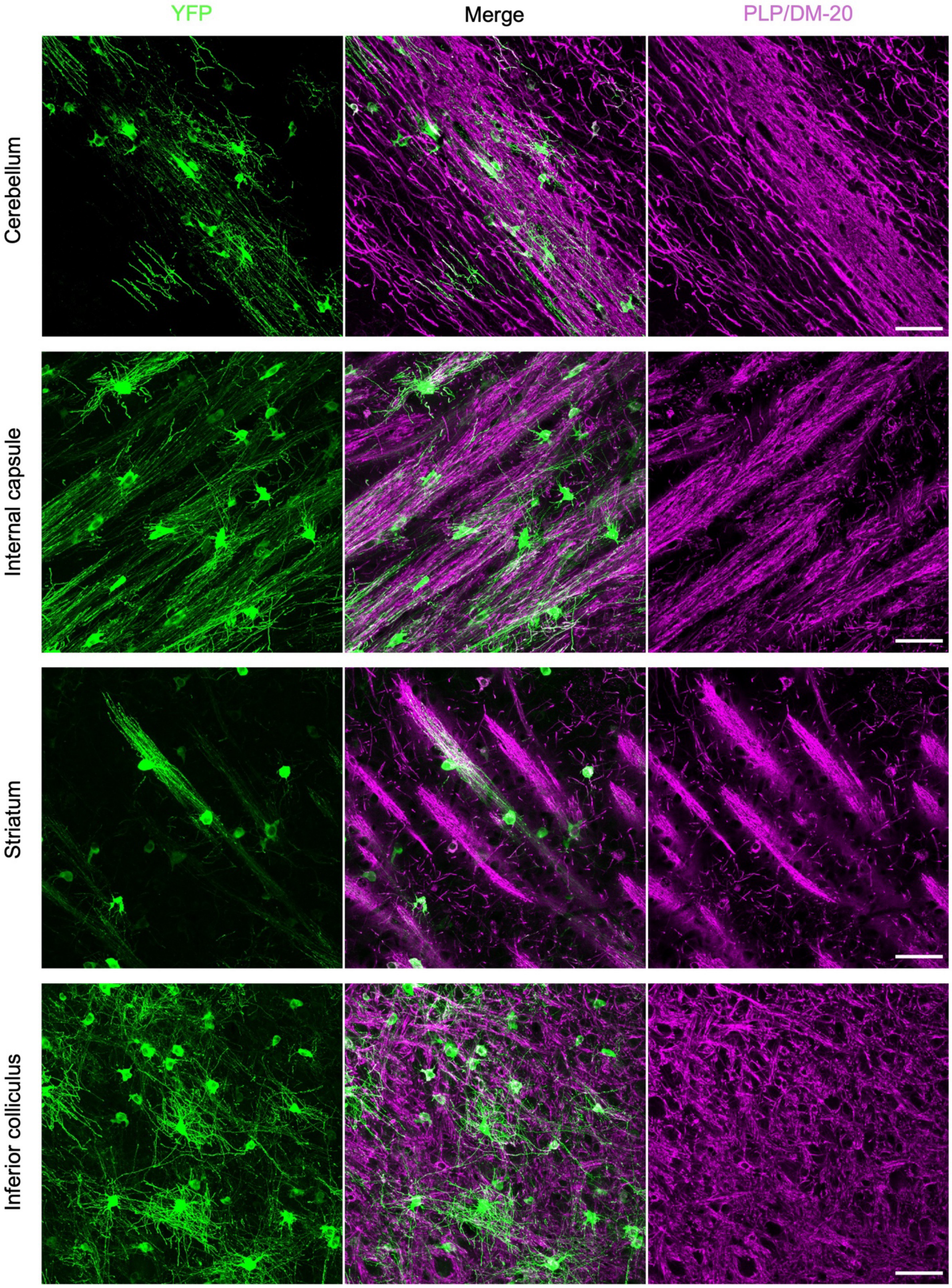

FIGURE S5 Yellow fluorescent protein (YFP)$^+$ oligodendrocytes (OLs) expressed proteolipid protein (PLP). YFP$^+$ OLs were observed throughout the central nervous system in P16 *LncOL1*-YFP mice. Scale bars, 50 μm.

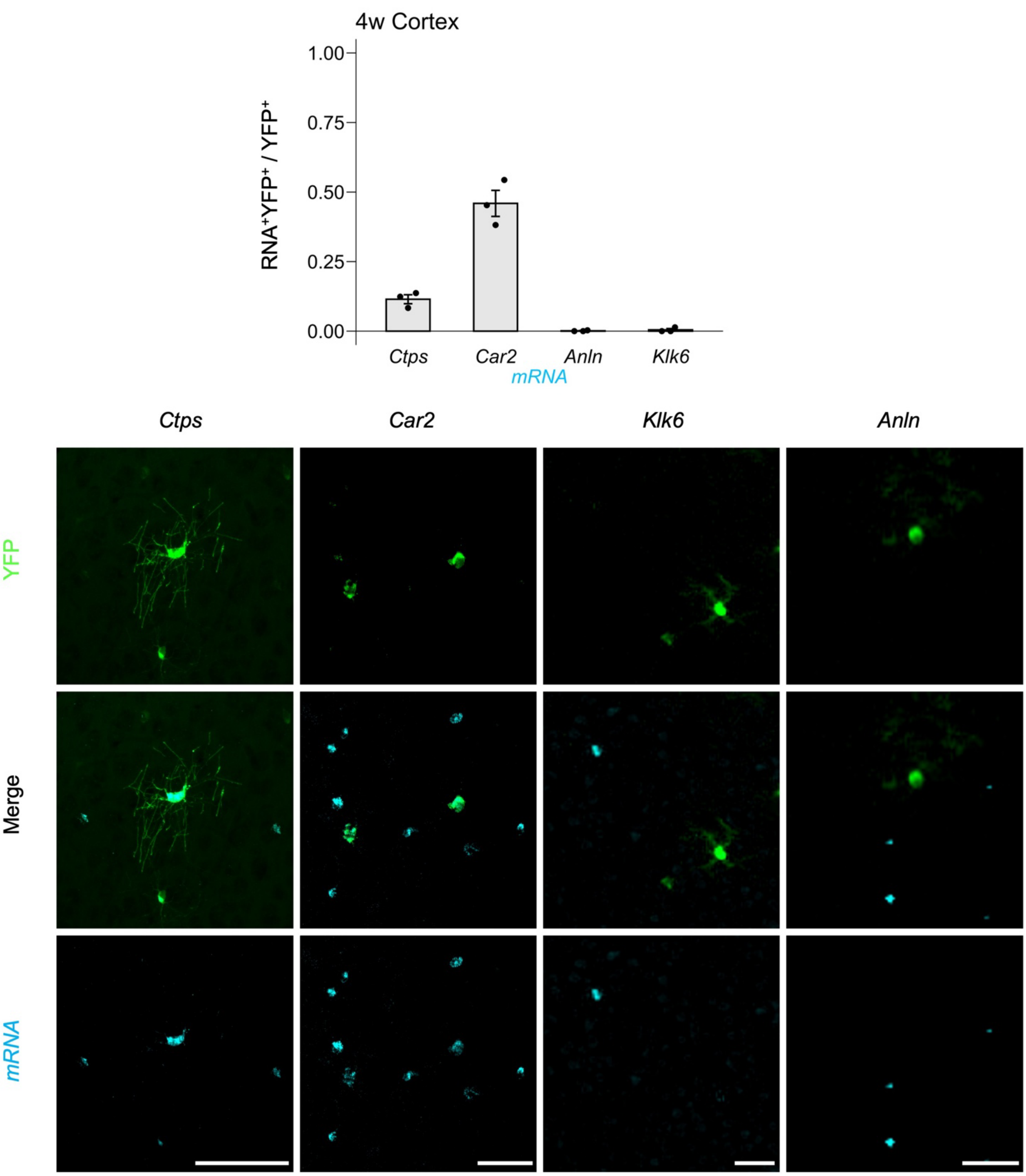

FIGURE S6 The yellow fluorescent protein (YFP)-expressing oligodendrocytes (OLs) were myelin-forming oligodendrocytes (MFOLs) in the postnatal week 4 long noncoding oligodendrocyte 1 gene (*LncOL1*)-YFP brain. The bar graph shows the ratio of the number of RNA marker-positive cells with YFP expression to the total YFP^+ cell number. *n* = 3 mice. The data are presented as the mean ± standard error of the mean. Scale bar, 50 μm.

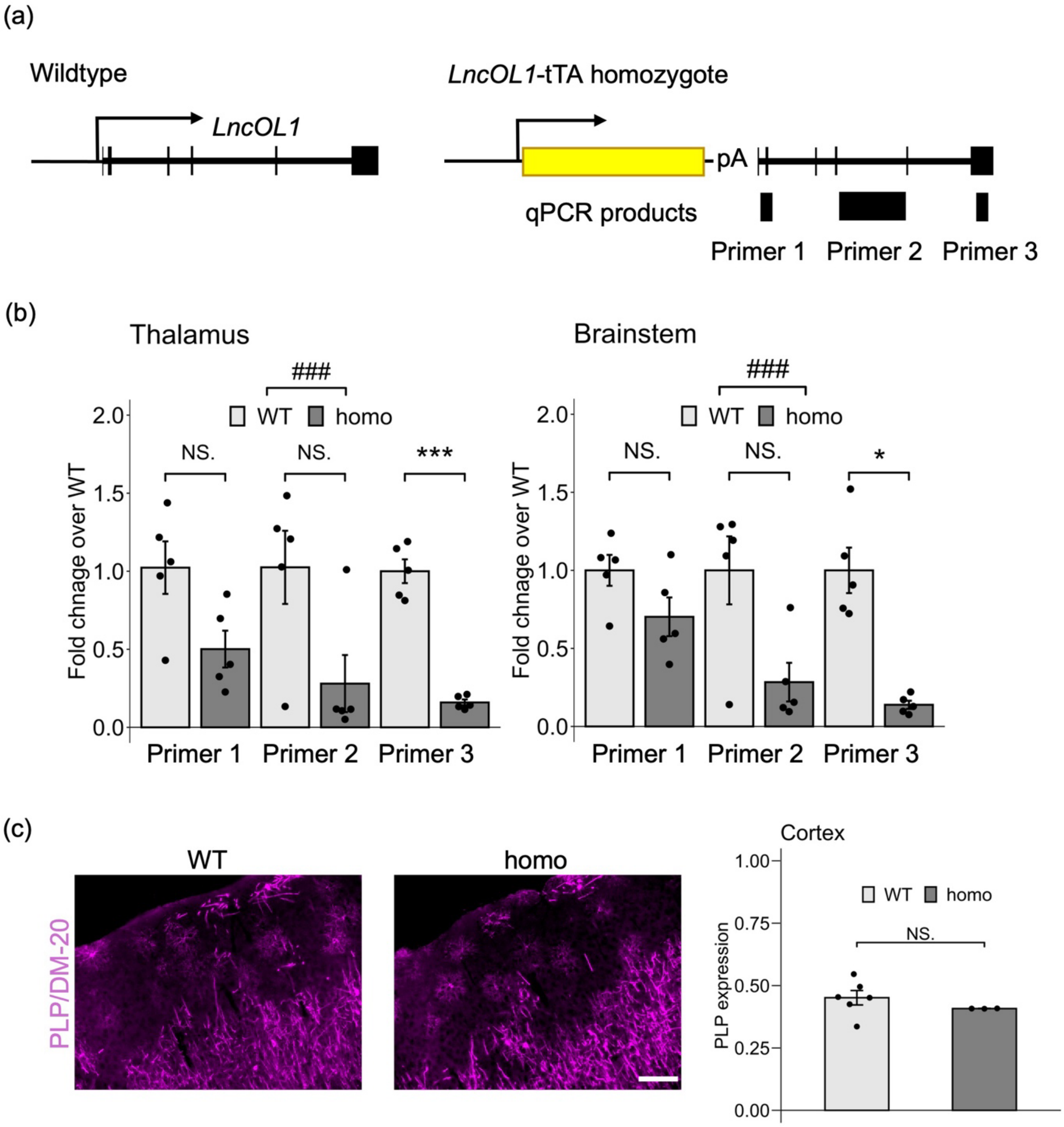

FIGURE S7 Long noncoding oligodendrocyte 1 gene (*LncOL1*) knockdown did not alter the proteolipid protein (PLP) immunoreactivity in the homozygous *LncOL1*-tTA mice. (a) Schematic illustration of the genomic constructs of the wild-type mice and homozygous *LncOL1*-tTA mice. In *LncOL1*-tetracycline transactivator (tTA) homozygous mice, downstream *LncOL1* is not transcribed if premature cleavage occurs at the polyA. qPCR primers were designed to be complementary sequences for each exon, as shown by the black box. (b) qPCR results. $n = 5$ mice for both the wild-type and *LncOL1*-tTA homozygous groups. A two-way repeated ANOVA was performed (genotype, $p < 0.001$ (###); primer, $p = 0.488$; interaction, $p = 0.564$ for the thalamus; genotype, $p < 0.001$ (###); primer, $p = 0.117$; interaction, $p = 0.117$ for the brainstem. Two-tailed unpaired t tests were performed to compare two genotypes within each primer set. $p < 0.05$ was considered the threshold for significance. The results are presented as *$p < 0.05$, **$p < 0.01$, ***$p < 0.001$ (Bonferroni corrected), and not significant (NS). (c) Representative images of PLP immunoreactivity in both wild-type and

LncOL1-tTA homozygous mouse brains. $n = 6$ wild-type mice and $n = 3$ *LncOL1*-tTA homozygous mice. Two-tailed unpaired t tests were performed to compare two groups. $p < 0.05$ was considered the threshold for significance. The results are presented as $^{*}p < 0.05$, $^{**}p < 0.01$, $^{***}p < 0.001$, and not significant (NS). n = number of independent brains. The bar graphs are presented as the mean ± standard error of the mean. Scale bar, 100 μm.

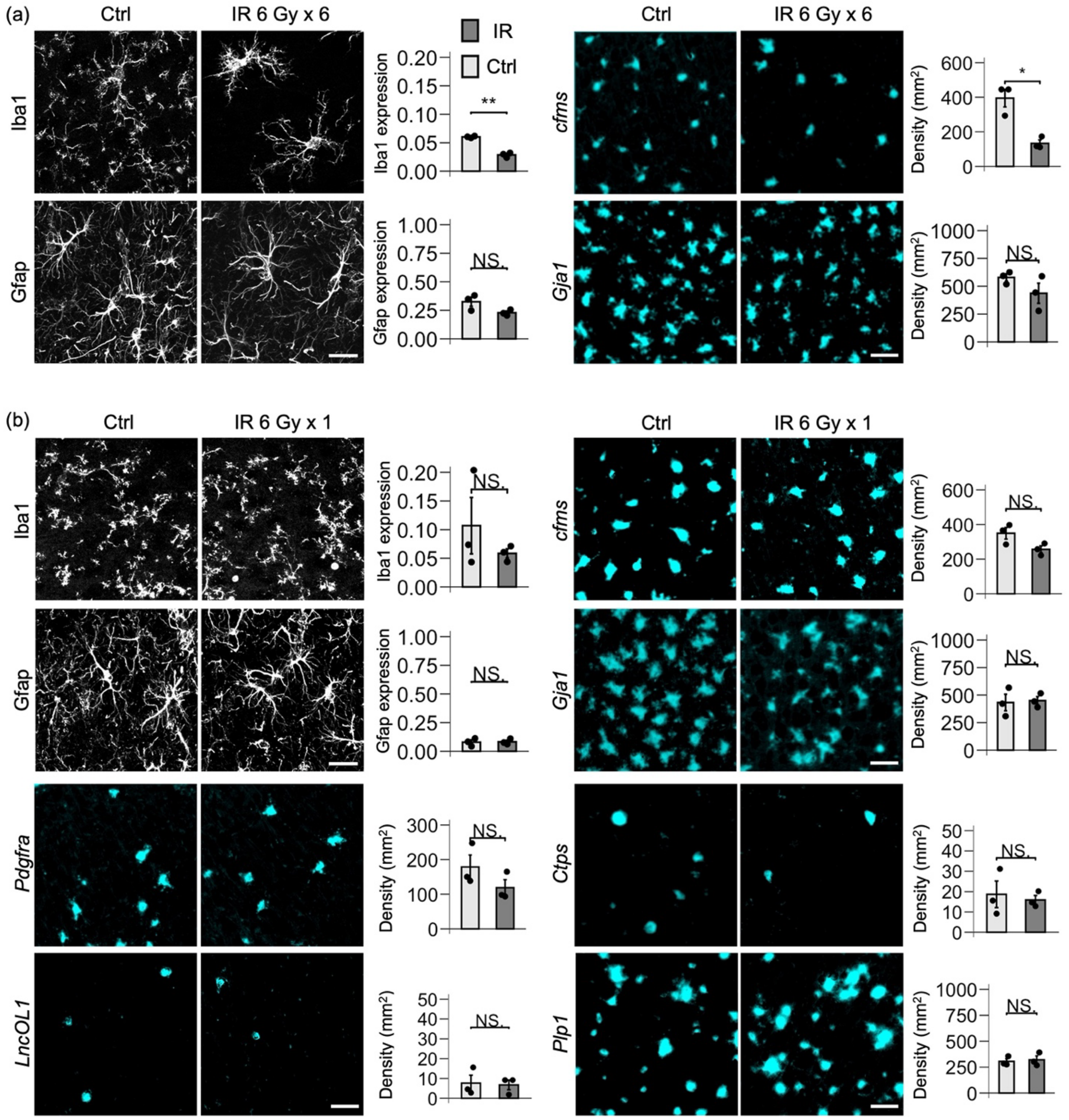

FIGURE S8 Glial response to the X-ray irradiation. (a) Microglia significantly decreased in number after 6 times of 6 Gy X-ray irradiation protocol in the Figure 6 (upper panels), quantified as Iba1$^+$ area and the number of *cfms* ISH signals. Astrocytes did not decrease significantly in number of *Gja1* ISH signals and area of Gfap expression. Scale bars, 20 µm for left panels, 50 µm for right panels. *n* = 3 mice. (b) Microglial and astrocytic acute reactivity was not detected in the 24 hours after single dose of 6 Gy X-ray irradiation (upper two rows). Scale bars, 20 µm for Iba1 and Gfap panels, 50 µm for other panels. X-ray irradiation did not acutely deplete oligodendrocyte populations in the course of differentiation (bottom two rows). Scale bars, 50 µm. *n* = 3 mice.

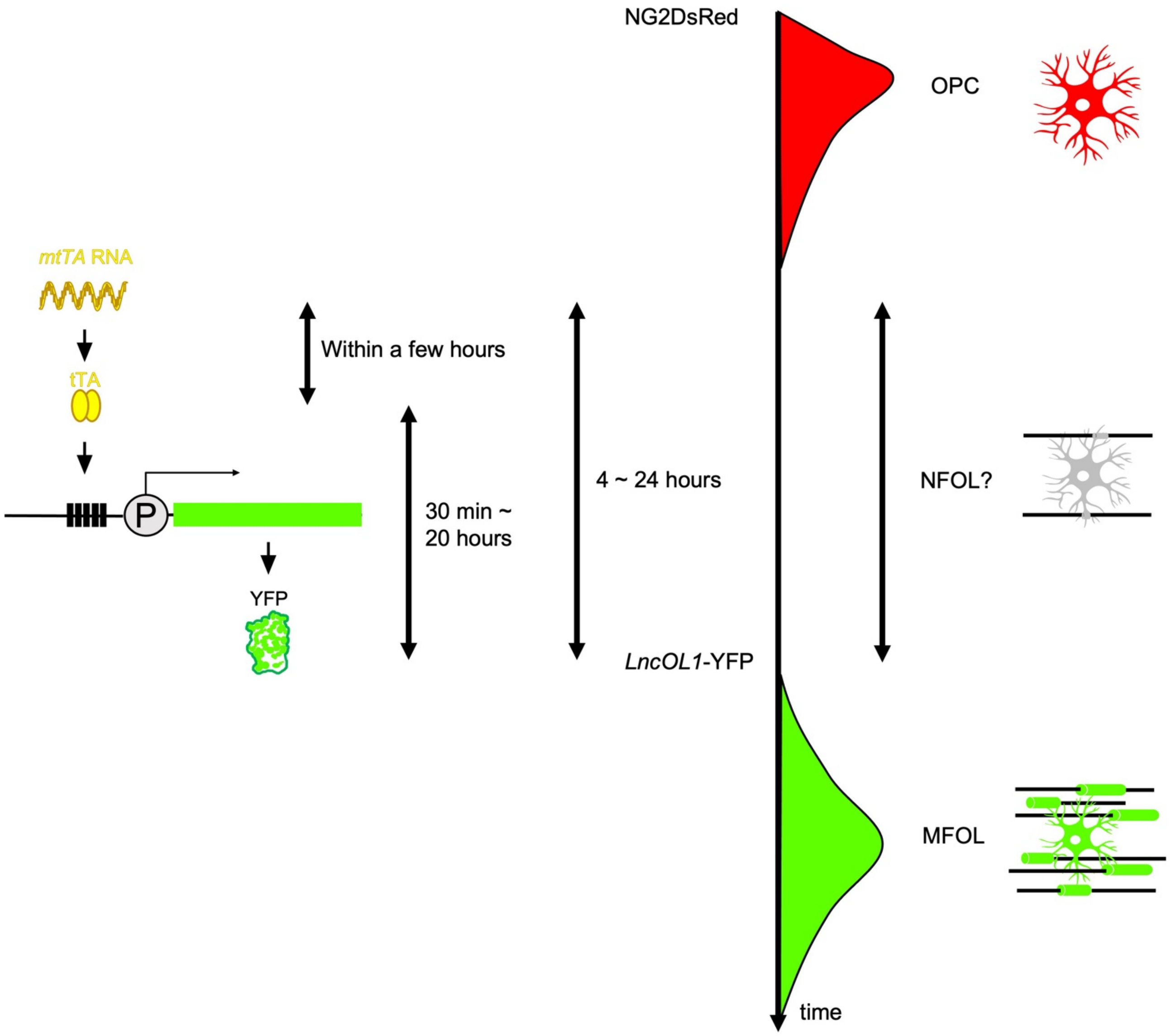

FIGURE S9 Schematic illustration of the time scale for yellow fluorescent protein (YFP) expression in the long noncoding oligodendrocyte 1 gene (*LncOL1*)-YFP mouse line. We estimated that the time required for YFP expression was from 4 to 24 h after *mtTA* mRNA transcription. To estimate the time for differentiating oligodendrocytes (OLs) to pass the newly formed oligodendrocyte (NFOL) phase, an experiment using the NG2DsRed line crossed with the *LncOL1*-YFP line can be performed. In that hypothetical experiment, the time between DsRed disappearance and YFP expression will indicate the duration for which the OLs exist as NFOLs.

TABLE S1 Statistic reports for Figure 6.

Normality assessment						
marker	group	result	statistical test	p-value		
Pdgfra	Ctrl	normally ditributed	shapiro test	0.3564		
Pdgfra	IR	normally ditributed	shapiro test	0.08916		
LncOL1	Ctrl	normally ditributed	shapiro test	0.2507		
LncOL1	IR	all zero values	-	-		
Ctps	Ctrl	normally ditributed	shapiro test	0.9969		
Ctps	IR	all zero values	-	-		
Plp1	Ctrl	not normally ditributed	shapiro test	0.005866		
Plp1	IR	normally ditributed	shapiro test	0.1921		
YFP	Ctrl	normally ditributed	shapiro test	0.6755		
YFP	IR	normally ditributed	shapiro test	0.1328		
Comparison						
marker	group	result	statistical test	p-value	t	df
Pdgfra	Ctrl vs IR	significant	Welch	0.003036	8.757	3.0252
LncOL1	Ctrl vs IR	significant	Welch	0.04446	3.3377	3
Ctps	Ctrl vs IR	significant	Welch	0.01554	4.9824	3
Plp1	Ctrl vs IR	not significant	Wilcoxon	0.2	W=13	
YFP	Ctrl vs IR	significant	Welch	0.01185	4.3401	4.0616

TABLE S2 Statistic reports for Figure S7(b).

Normality assessment							
region	group	result	statistical test	p-value			
thalamus	WT P1	normally ditributed	shapiro test	0.6713			
thalamus	homo P1	normally ditributed	shapiro test	0.5357			
thalamus	WT P2	normally ditributed	shapiro test	0.1451			
thalamus	homo P2	not normally ditributed	shapiro test	0.000907			
thalamus	WT P3	normally ditributed	shapiro test	0.4126			
thalamus	homo P3	normally ditributed	shapiro test	0.366			
brainstem	WT P1	normally ditributed	shapiro test	0.4602			
brainstem	homo P1	normally ditributed	shapiro test	0.7076			
brainstem	WT P2	not normally ditributed	shapiro test	0.008358			
brainstem	homo P2	not normally ditributed	shapiro test	0.03727			
brainstem	WT P3	normally ditributed	shapiro test	0.3113			
brainstem	homo P3	normally ditributed	shapiro test	0.8027			
ANOVA							
Thalamus							
Source of Variation	*SS*	*df*	*MS*	*F*	*P-value*	*F crit*	*result*
Sample (primer)	0.168442802	2	0.084221401	0.73862442	0.48832005	3.40282611	not signifiacnt among primers
Columns (genotype)	3.701085282	1	3.701085282	32.4586381	7.2053E-06	4.25967727	significant between genotypes
Interaction	0.133482146	2	0.066741073	0.58532138	0.56468107	3.40282611	not significant interaction
Within	2.736591921	24	0.114024663				
Total	6.739602151	29					
Brainstem							
Source of Variation	*SS*	*df*	*MS*	*F*	*P-value*	*F crit*	*result*
Sample	0.428509746	2	0.214254873	2.34012487	0.11790705	3.40282611	not signifiacnt among primers
Columns	2.929503139	1	2.929503139	31.9964865	7.9737E-06	4.25967727	significant between genotypes
Interaction	0.428509747	2	0.214254873	2.34012487	0.11790705	3.40282611	not significant interaction
Within	2.197368621	24	0.091557026				
Total	5.983891252	29					
region	comparison	result	statistical test	bonferroni corrected p-value			
thalamus	WT P1 vs homo P1	not significant	Welch	0.0759			
thalamus	WT P2 vs homo P2	not significant	Wilcoxon	0.0759			
thalamus	WT P3 vs homo P3	significant	Welch	0.0007			
brainstem	WT P1 vs homo P1	not significant	Welch	0.0993			
brainstem	WT P2 vs homo P2	not significant	Wilcoxon	0.0544			
brainstem	WT P3 vs homo P3	significant	Welch	0.0107			

TABLE S3 Statistic reports for Figure S7(c).

Normality assessment					
group	result	statistical test	p-value		
WT	noramally ditributed	shapiro test	0.8544		
homo	noramally ditributed	shapiro test	1		
Comparison					
group	result	statistical test	p-value	t	df
WT vs homo	not significant	Welch	0.1926	1.5051	5.0018

TABLE S4 Statistic reports for Figure S8(a).

Normality assessment						
marker	group	result	statistical test	p-value		
gfap	Ctrl	normally ditributed	shapiro test	0.4567		
gfap	IR	normally ditributed	shapiro test	0.7391		
Iba1	Ctrl	normally ditributed	shapiro test	0.7997		
Iba1	IR	normally ditributed	shapiro test	0.503		
gja1	Ctrl	normally ditributed	shapiro test	0.5684		
gja1	IR	normally ditributed	shapiro test	0.9328		
cfms	Ctrl	not normally ditributed	shapiro test	< 2.2e-16		
cfms	IR	normally ditributed	shapiro test	0.1597		
Comparison						
marker	group	result	statistical test	p-value	t	df
gfap	Ctrl vs IR	not significant	Welch	0.1251	2.268	2.5149
Iba1	Ctrl vs IR	significant	Welch	0.002628	12.113	2.5312
Gja1	Ctrl vs IR	not significant	Welch	0.2507	1.4895	2.4916
cfms	Ctrl vs IR	significant	Welch	0.02379	4.8057	2.5913

TABLE S5 Statistic reports for Figure S8(b).

Normality assessment						
marker	group	result	statistical test	p-value		
gfap	Ctrl	normally ditributed	shapiro test	0.8431		
gfap	IR	normally ditributed	shapiro test	0.5352		
Iba1	Ctrl	normally ditributed	shapiro test	0.3412		
Iba1	IR	normally ditributed	shapiro test	0.7566		
Gja1	Ctrl	normally ditributed	shapiro test	0.8639		
Gja1	IR	normally ditributed	shapiro test	0.8247		
cfms	Ctrl	normally ditributed	shapiro test	0.5222		
cfms	IR	normally ditributed	shapiro test	0.9559		
Pdgfra	Ctrl	normally ditributed	shapiro test	0.1905		
Pdgfra	IR	normally ditributed	shapiro test	0.1094		
LncOL1	Ctrl	normally ditributed	shapiro test	0.253		
LncOL1	IR	not normally ditributed	shapiro test	< 2.2e-16		
Ctps	Ctrl	normally ditributed	shapiro test	0.5491		
Ctps	IR	normally ditributed	shapiro test	0.4633		
Plp1	Ctrl	normally ditributed	shapiro test	0.201		
Plp1	IR	normally ditributed	shapiro test	0.5433		
Comparison						
marker	group	result	statistical test	p-value	t	df
gfap	Ctrl vs IR	not significant	Welch	0.8968	-0.13911	3.6048
Iba1	Ctrl vs IR	not significant	Welch	0.4299	0.97019	2.1044
Gja1	Ctrl vs IR	not significant	Welch	0.8513	-0.20479	2.9029
cfms	Ctrl vs IR	not significant	Welch	0.09008	2.387	3.2609
Pdgfra	Ctrl vs IR	not significant	Welch	0.2329	1.4409	3.4911
LncOL1	Ctrl vs IR	not significant	Welch	0.8566	0.19528	3.3074
Ctps	Ctrl vs IR	not significant	Welch	0.7221	0.39892	2.4493
Plp1	Ctrl vs IR	not significant	Welch	0.7512	-0.34276	3.5388
